# Morphological Staging and Transcriptomic Analyses Reveal Divergent Reproductive Organ Fates in Papaya Flowers

**DOI:** 10.3390/plants15162530

**Published:** 2026-08-21

**Authors:** Lijian Zhang, Yutong Zheng, Zhihui Yang, Yihan Tong, Mengjun Xiao, Ray Ming

**Affiliations:** 1 College of Life Sciences, Fujian Agriculture and Forestry University, Fuzhou 350002, China; zlj18270162083@163.com (L.Z.); t13476018526@gmail.com (Y.T.); 2 Center for Genomics and Biotechnology, Fujian Provincial Key Laboratory of Haixia Applied Plant Systems Biology, Key Laboratory of Genetics, Breeding and Multiple Utilization of Crops, Ministry of Education, Fujian Agriculture and Forestry University, Fuzhou 350002, China

**Keywords:** *Carica papaya*, floral development, hermaphroditic flower, pistil abortion, sexual differentiation

## Abstract

Papaya is a trioecious species, but a unified framework for comparing floral sex differentiation has been lacking. We combined stereomicroscopy, paraffin sectioning, and scanning electron microscopy to establish a continuous 12-stage developmental system from floral primordium initiation to anthesis. Male, female, and hermaphroditic flowers followed a common developmental trajectory during Stages 1–4 and diverged at Stage 5. Male flowers formed fertile stamens and a reduced pistillode, female flowers developed a complete gynoecium without stamens, and hermaphroditic flowers displayed normal, aborted, or transitional pistils and carpellodic stamens. RNA sequencing and weighted gene co-expression network analysis of morphologically defined tissues identified gene modules associated with normal pistil development, distinct modes of pistil abortion, and stamen carpellody. These modules were enriched in chromatin and RNA regulation, protein homeostasis, floral-organ identity, hormone and receptor-kinase signaling, pollen-wall formation, and stress-responsive pathways, yielding 30 representative candidate genes. This developmental framework integrates developmental morphology with transcriptional programs underlying reproductive-organ fate and plasticity in papaya.

## 1. Introduction

Papaya (*Carica papaya* L.) is an economically important tropical and subtropical fruit crop valued for its rapid growth, early fruiting, high productivity, and nutritional quality [1]. It is also a useful system for studying plant sex determination because it has male, female, and hermaphroditic plants. Sex in papaya is controlled by a recently evolved sex-chromosome system: female plants are XX, male plants are XY, and hermaphroditic plants are XYh [2,3,4,5]. These sex types differ strongly in reproductive organ development. Male flowers develop fertile stamens and a reduced pistillode, female flowers form a functional gynoecium but generally lack fertile stamens, and hermaphroditic flowers contain both stamens and a central pistil [6]. The pistil of hermaphroditic flowers is particularly variable, ranging from normally developed fertile gynoecia to aborted, partially recovered, or carpellodic structures. This plasticity makes papaya suitable for examining how floral organ identity, pistil development, organ abortion, and sexual fate are coordinated during floral morphogenesis.

Flower development is a continuous process that includes floral meristem initiation, sepal and petal primordium formation, stamen and carpel differentiation, gametophyte development, and anthesis [7]. These events are reflected in both external bud morphology and internal anatomical changes. A reproducible staging system is therefore essential for integrating morphology with gene expression, hormone regulation, and organ identity specification. Such staging systems have been widely used in *Arabidopsis*, tomato, and cucumber to connect morphological landmarks with molecular regulation and reproductive organ arrest [8,9,10,11]. Staging is especially important in species with unisexual or complex sexual systems. Sex differentiation often does not begin as a complete absence of an organ; instead, it involves the promotion, suppression, arrest, or abortion of specific reproductive organs within defined developmental windows [8,12]. Therefore, determining the timing and morphological characteristics of these developmental divergence points is a prerequisite for understanding the regulatory mechanisms underlying sex expression. For example, in cucumber, both stamen arrest in female flowers and pistil arrest in male flowers occur at specific developmental stages and are closely associated with the expression of floral organ identity genes and hormone-related pathways [13]. These studies indicate that a floral staging system is not merely a descriptive tool for morphological changes, but also a necessary temporal framework for interpreting genetic regulation, hormonal signaling, and transcriptomic dynamics during reproductive organ development.

Early morphological and anatomical studies provided the first detailed description of floral organogenesis in papaya and showed that staminate and pistillate flowers follow similar developmental trajectories during early organ initiation before diverging during reproductive organ differentiation [6]. Subsequent genetic and genomic studies established that papaya sex is controlled by a recently evolved sex-chromosome system and identified sex-specific genomic regions and candidate genes associated with male and hermaphroditic development [2,3,4]. In addition to genetic control, papaya sex expression is developmentally plastic. Low-temperature-induced sex reversal, sex-biased hormone-related gene expression, and differences in DNA methylation have indicated that environmental signals, phytohormone pathways, and epigenetic regulation may modify reproductive organ development within the constraints imposed by sex genotype [14,15,16]. More recent transcriptomic and gene-regulatory network analyses have further identified developmental stage- and organ-specific expression patterns associated with normal pistil development, pistil abortion, and stamen differentiation, with prominent contributions from hormone signaling, floral organ identity factors, and transcriptional regulatory networks [17,18,19].

In this study, we established a continuous 12-stage developmental framework for male, female, and hermaphroditic papaya flowers using stereomicroscopy, paraffin sectioning, and scanning electron microscopy. We first identified the developmental stage at which the three floral morphs began to diverge and characterized the subsequent trajectories of normal pistil development, pistil abortion, transitional pistil development, and stamen carpellody. These morphologically defined organ states were then used to guide tissue sampling for RNA sequencing. By integrating developmental staging with tissue-resolved transcriptomic and co-expression analyses, we aimed to identify candidate gene modules associated with divergent reproductive organ fates in papaya. Male and female flowers were sampled from the cultivar AU9, whereas hermaphroditic flowers were sampled from the cultivar Zhongbai (ZB). Therefore, comparisons involving hermaphroditic flowers may reflect both sex-type and cultivar-specific effects, and the resulting transcriptional associations require validation across common or additional genetic backgrounds.

## 2. Results

### 2.1. Floral Structure of Papaya

**Figure 1:**
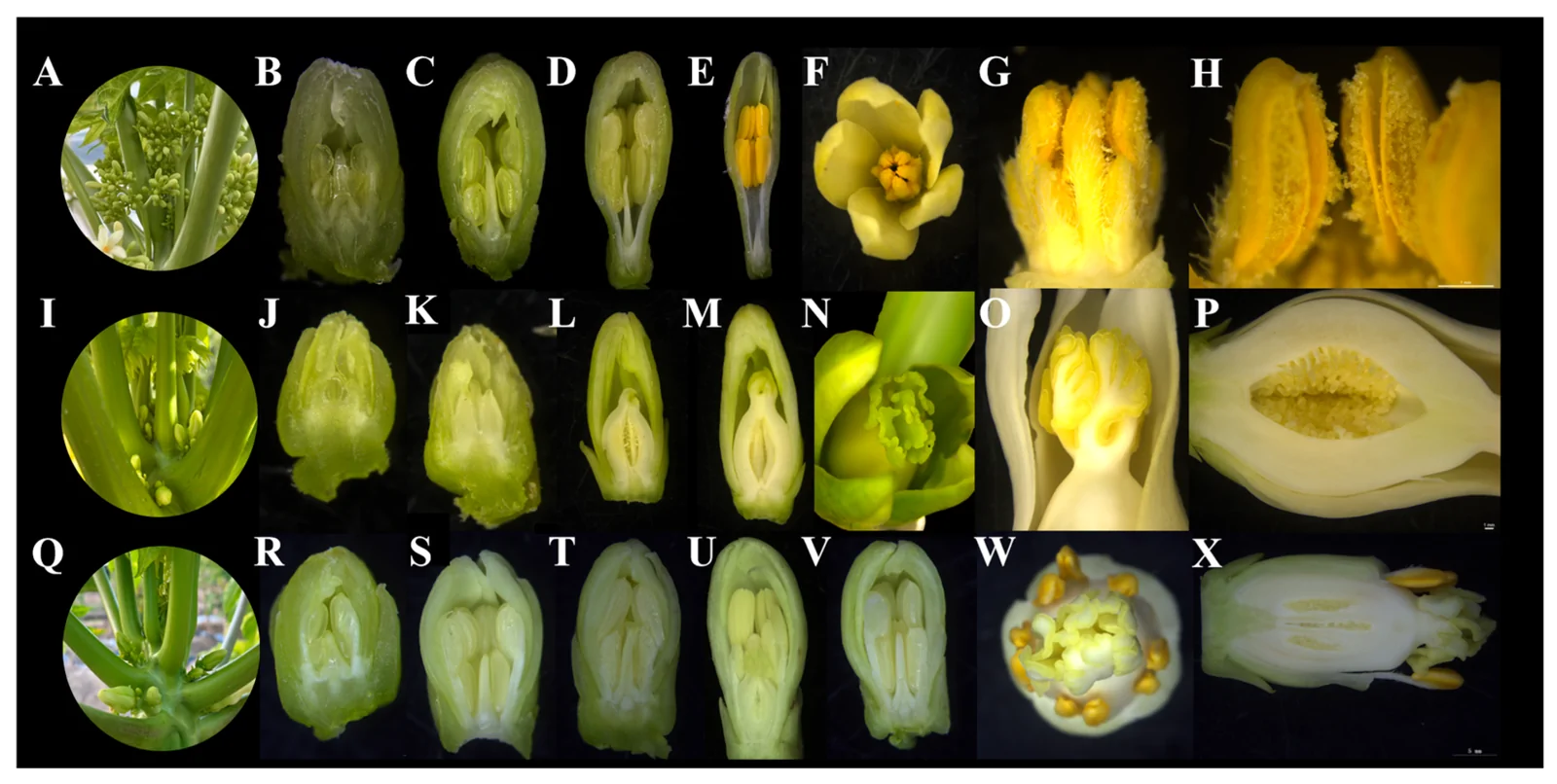
Floral structures of male, female, and hermaphroditic papaya flowers. (**A**) Natural appearance of male flowers; (**B**) longitudinal section of a 1 mm male flower bud; (**C**) longitudinal section of a 2.5 mm male flower bud; (**D**) longitudinal section of a 6 mm male flower bud; (**E**) longitudinal section of a 12 mm male flower bud; (**F**) corolla structure of a male flower; (**G**) structure of mature stamens in a male flower; (**H**) anther dehiscence in a male flower; (**I**) natural appearance of a female flower, Scale bar = 1 mm.; (**J**) longitudinal section of a 1 mm female flower bud; (**K**) longitudinal section of a 2 mm female flower bud; (**L**) longitudinal section of a 6 mm female flower bud; (**M**) longitudinal section of a 10 mm female flower bud; (**N**) corolla structure of a female flower; (**O**) stigma structure of a female flower; (**P**) ovary locule structure of a female flower, Scale bar = 1 mm; (**Q**) natural appearance of a hermaphroditic flower; (**R**) longitudinal section of a 1 mm hermaphroditic flower bud; (**S**) longitudinal section of a 2 mm hermaphroditic flower bud; (**T**) longitudinal section of a 5 mm hermaphroditic flower bud; (**U**) longitudinal section of an 8 mm hermaphroditic flower bud with a fertile pistil; (**V**) longitudinal section of an 8 mm hermaphroditic flower bud with an aborted pistil; (**W**) pistil and stamen structures of a fertile hermaphroditic flower; (**X**) ovary locule structure of a fertile hermaphroditic flower, Scale bar = 5 mm.

The morphological and transcriptomic comparisons included male and female flowers from the cultivar AU9 and hermaphroditic flowers from the cultivar Zhongbai (ZB). Papaya flowers exhibited distinct morphological differences among male, female, and hermaphroditic sex types (Figure 1). All three flower types were generally actinomorphic and pentamerous, with five sepals and five petals. However, the degree of petal fusion, corolla shape, stamen development, and pistil development differed markedly among the three sex types.

Male flowers were borne on long, highly branched inflorescences with a slender and tubular morphology (Figure 1A). Their petals were fused at the base to form an elongated corolla tube (Figure 1E). Ten stamens were arranged in two whorls within the corolla tube (Figure 1F), and mature anthers dehisced to release pollen (Figure 1G,H). In contrast, the central pistil was reduced to a small pistillode lacking a normal functional stigma, ovary cavity, and ovules (Figure 1B–E). Thus, male flowers were functionally characterized by fertile stamens and arrested pistil development.

Female flowers were usually solitary or borne in small clusters on short inflorescences (Figure 1I). They were larger than male flowers and possessed a more open perianth, with petals that were nearly free or only slightly fused at the base (Figure 1J–M). The most distinctive feature was a fully developed gynoecium. The enlarged ovary enclosed a well-defined ovarian cavity containing numerous ovules attached to parietal placentae (Figure 1L,M,P), while the stigma was divided into five lobes that expanded into fan-shaped, petaloid, or irregularly lobed structures at maturity (Figure 1O). Female flowers lacked any traces of stamens, and following successful pollination and fertilization, female flowers developed into fleshy fruits.

Hermaphroditic flowers combined male and female characteristics and exhibited remarkable plasticity in pistil development (Figure 1R–X). They were usually borne singly or on short inflorescences, and individual flowers were generally slender, tubular, or pear-shaped because of basal petal fusion (Figure 1Q). Most hermaphroditic flowers developed ten fertile stamens comparable to those of male flowers, together with a central pistil that displayed diverse developmental outcomes (Figure 1R–V). Some flowers produced a fully functional pistil with a well-developed ovary, ovarian cavity, ovules, and a receptive stigma (Figure 1U,X), whereas others formed a severely reduced, aborted pistil resembling the male pistillode (Figure 1V). These observations indicate that pistil development in hermaphroditic flowers varies continuously from normal development to partial development and abortion.

### 2.2. Developmental Staging and Sex Differentiation of Papaya Flowers

Based on the continuous morphological changes from floral primordium initiation to anthesis, papaya floral development was divided into 12 stages. The three floral morphs followed a common developmental trajectory during Stages 1–4, encompassing inflorescence meristem activation, floral primordium formation, sepal primordium initiation, and petal primordium differentiation. Visible sexual differentiation first became apparent at Stage 5.

**Figure 2:**
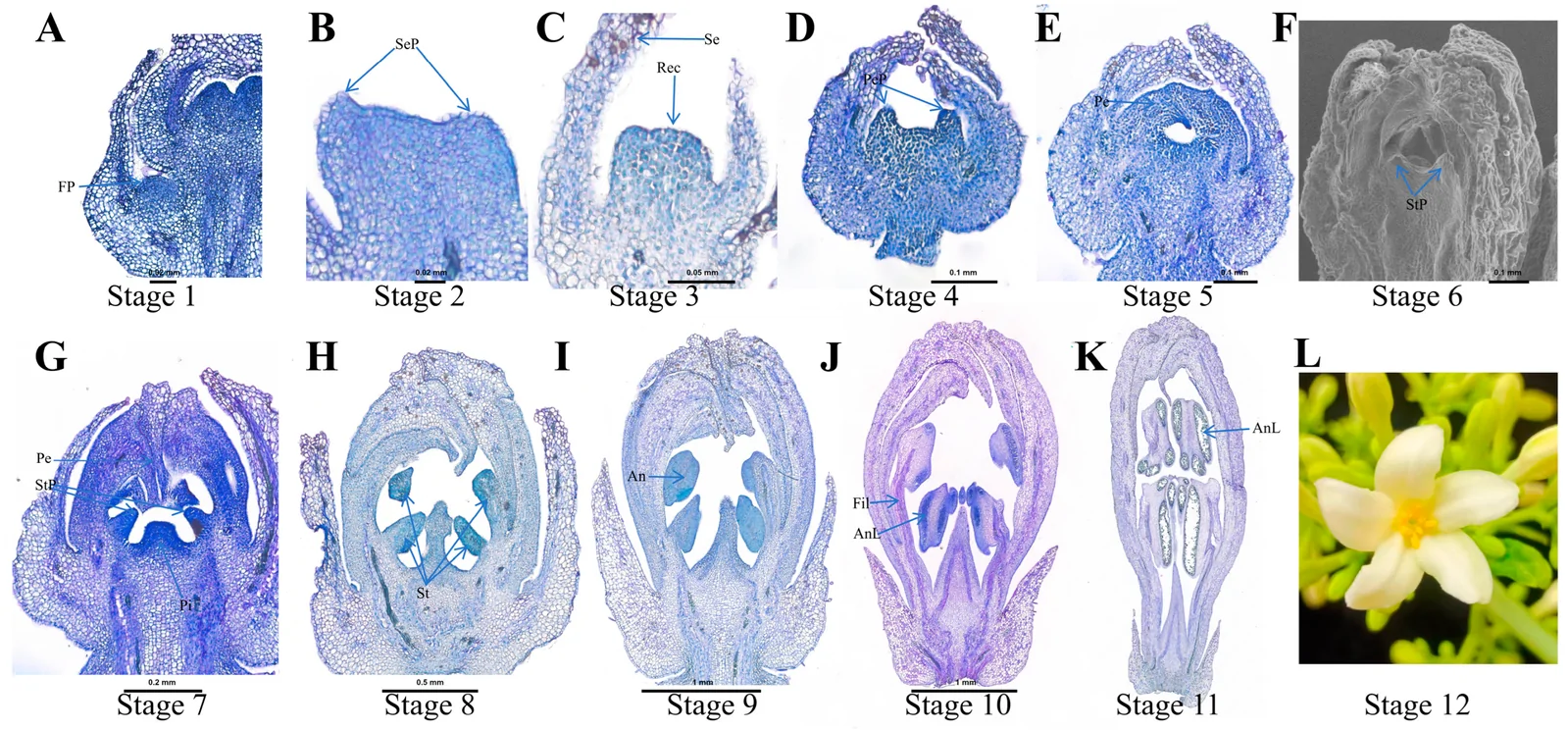
Paraffin sections of AU9 male papaya flowers at different developmental stages. Abbreviations: FP, floral primordium; SeP, sepal primordium; PeP, petal primordium; Se, sepal; Pe, petal; Pi, pistil; Sg, stigma; StP, stamen primordium; St, stamen; An, anther; AnL, anther locule; Fil, filament; Rec, receptacle. (**A**) The floral primordium began to protrude from the floral receptacle, Scale bar = 0.02 mm. (**B**) Sepal primordia began to protrude from the floral receptacle. (**C**) Initial completion of sepal development. (**D**) Five petal primordia continued to enlarge and became clearly distinguishable. (**E**) Structural differentiation of the petal primordia was initiated. (**F**) SEM of the stamen primordia. (**G**) Petal structures were nearly fully developed; the central carpel primordia rapidly emerged and fused, while the antesepalous stamen primordia were initiated. (**H**) The ante-petalous stamen primordia appeared and rapidly developed into stalk-like structures. (**I**) The two whorls of stamens developed synchronously, and early anther locule structures became visible. (**J**) The anther locules further differentiated, and pollen mother cells began to form. (**K**) The anther locules reached maturity. (**L**) A fully opened male flower.

At Stage 1, the inflorescence meristem was activated in the upper leaf axil and gave rise to a young cyme. Distinct floral primordium bulges were visible on the flanks of the inflorescence meristem. Male inflorescences were generally larger and more highly branched than female or hermaphrodite inflorescences (Figure 2A). At Stage 2, hemispherical floral primordia emerged and became separated from the inflorescence meristem by shallow grooves. The basal region of each floral primordium underwent slight longitudinal elongation, while sepal primordia were initiated successively (Figure 2B). At Stage 3, five sepal primordia were fully established and elongated rapidly, enclosing the inner floral primordium in an imbricate arrangement (Supplementary Figure S1A,B). As the sepals expanded, trichomes and glandular hairs became clearly visible on their abaxial surfaces (Supplementary Figure S1G). Meanwhile, a raised floral receptacle developed in the central region (Figure 2C; Supplementary Figure S1B). At Stage 4, five petal primordia were initiated almost simultaneously and began to display an early contorted arrangement. Concurrently, the central carpel-forming region became progressively thickened. The petal primordia continued to elongate into stalk-like structures and subsequently differentiated into rudimentary petals, marking the completion of Stage 4 (Figure 2D). No discernible morphological differences were observed among the three floral morphs before Stage 5. From Stage 5 onward, the three floral morphs followed distinct developmental trajectories. At Stage 5, in male flowers, rapid petal elongation and outward expansion of the corolla base initiated corolla tube formation (Figure 2E). At Stage 6, five outer-whorl stamen primordia, positioned opposite the sepals, began to emerge, whereas the central carpel primordia were not yet clearly distinguishable (Figure 2F). At Stage 7, the stamen primordia continued to elongate, while five carpel primordia became visible in the central region and developed rapidly. These carpel primordia subsequently fused to form a spear-shaped sterile pistillode, which persisted during later development but failed to differentiate into a functional pistil (Figure 2G). At Stage 8, the outer-whorl stamen primordia enlarged and developed into rod-like structures (Supplementary Figure S1C,I), and five inner-whorl stamen primordia positioned opposite the petals began to emerge. The outer-whorl stamens were longer than the inner-whorl stamens. Meanwhile, the basal regions of the stamens proliferated coordinately with the fused petal tissues (Figure 2H; Supplementary Figure S1J). At Stage 9, the outer- and inner-whorl stamens underwent rapid elongation and differentiation, forming complete stamens with clearly distinguishable anthers and filaments. However, distinct anther locule structures were not yet observed at this stage (Figure 2I; Supplementary Figure S1D). At Stage 10, the anthers matured further, with locule wall thickening, calcium oxalate crystal accumulation, and the onset of pollen development. The corolla tube had reached approximately two-thirds of its final length (Figure 2J; Supplementary Figure S1D). At Stage 11, pollen mother cells underwent meiosis and produced pollen grains. The pollen wall gradually developed, and the corolla tube elongated rapidly toward its final length (Figure 2K; Supplementary Figure S2E,F). At Stage 12, male flowers reached anthesis. The lobes of the long tubular corolla reflexed outward. The anthers dehisced and released pollen (Figure 2L).

**Figure 3:**
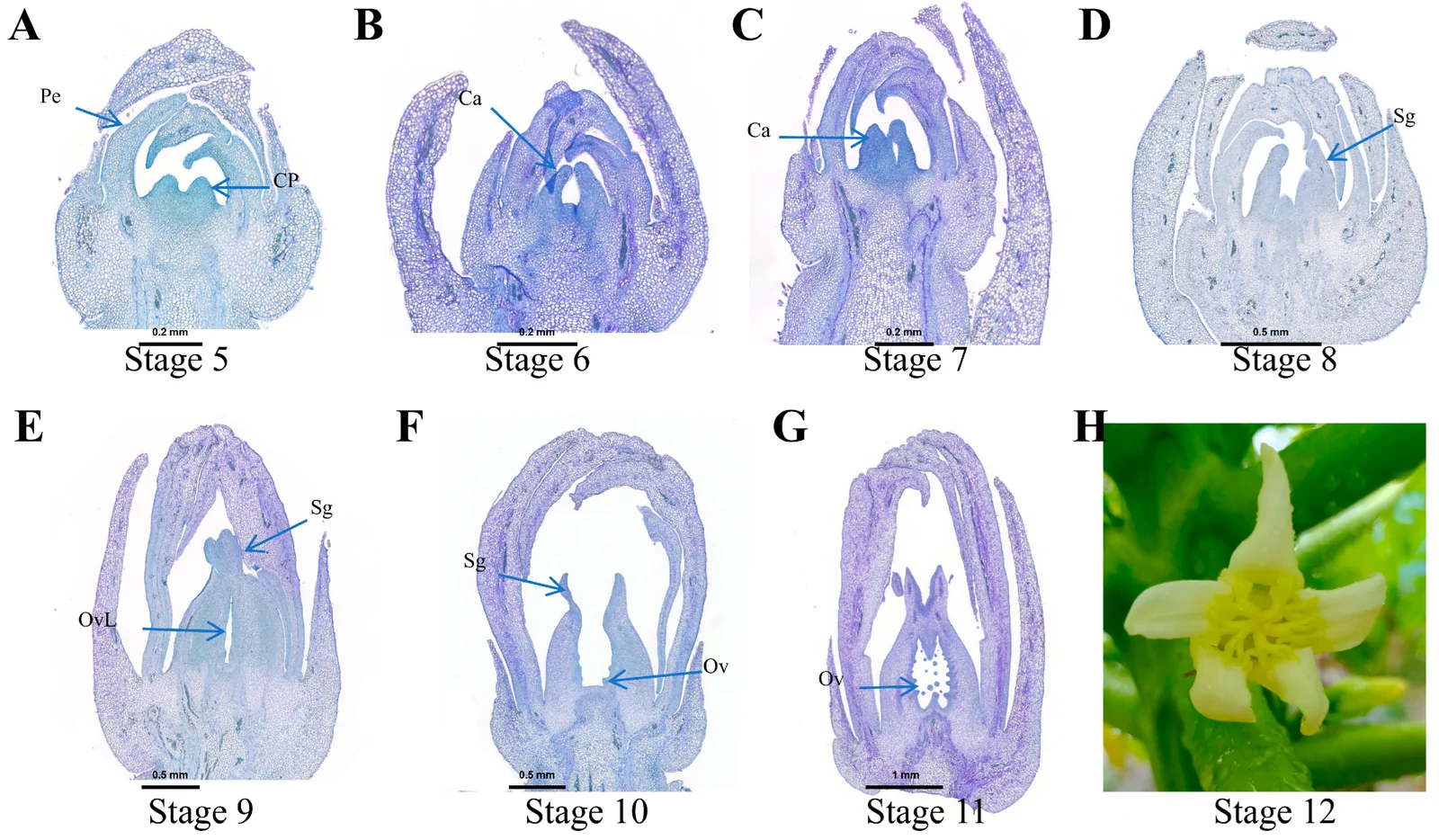
Histological characterization of AU9 female papaya flowers at different developmental stages. Abbreviations: PeP, petal primordium; Pe, petal; CP, carpel primordium; Ca, carpel; Pi, pistil; Sg, stigma; Ov, ovule; OvL, ovary locule. (**A**) Nearly complete petal development and initiation of five carpel primordia. (**B**) Initial fusion of the apical regions of the carpel primordia. (**C**) Outward growth of the carpel apices and their initial differentiation into discoid structures. (**D**) Further development of carpels and gradual approximation of adjacent carpels. (**E**) Complete fusion of carpels to form ovary locules, accompanied by stigma differentiation at the apex. (**F**) Initial formation of ovules within the ovary locules. (**G**) Formation of numerous ovules and full maturation of the stigma with mucilage secretion. (**H**) A female flower at anthesis.

Female flowers showed a different developmental pattern. Before Stage 5, the initiation and development of petal primordia in female flowers were consistent with those observed in male flowers (Figure 3A; Supplementary Figure S2A,B). After the onset of sexual differentiation, no stamen primordia were observed. At Stage 5, the five carpel primordia enlarged markedly and became clearly defined (Figure 3A). The petals continued to expand, but no tubular structure associated with stamens was formed. At Stage 6, the carpel margins grew inward and fused with each other. This process formed a closed ovary wall, and the ovary showed a pentagonal outline (Figure 3B; Supplementary Figure S2F). At Stage 7, the septa extended further inward, dividing the ovary into five developing locules, while the placental tissue continued to enlarge (Figure 3C). At Stage 8, the carpel apex expanded to form globular or finger-like stigma primordia. Specialized stigmatic tissues began to differentiate (Figure 3D; Supplementary Figure S2G). At Stage 9, the stigma lobes developed further to form the typical antler-like stigma of papaya female flowers. The ovary locules were fully differentiated, the placenta was well developed, and the overall pistil structure was nearly mature (Figure 3E; Supplementary Figure S2D,H). At Stage 10, numerous anatropous ovules were developed on the placenta. The internal structures of the ovules began to differentiate (Figure 3F; Supplementary Figure S2E). At Stage 11, the ovules continued to develop, and the integuments thickened. The pistil reached a mature, receptive state (Figure 3G). At Stage 12, the female flowers opened, and the petals expanded outward. The stigma lobes secreted abundant viscous exudates, indicating that the female flowers had reached full receptivity for fertilization (Figure 3H).

**Figure 4:**
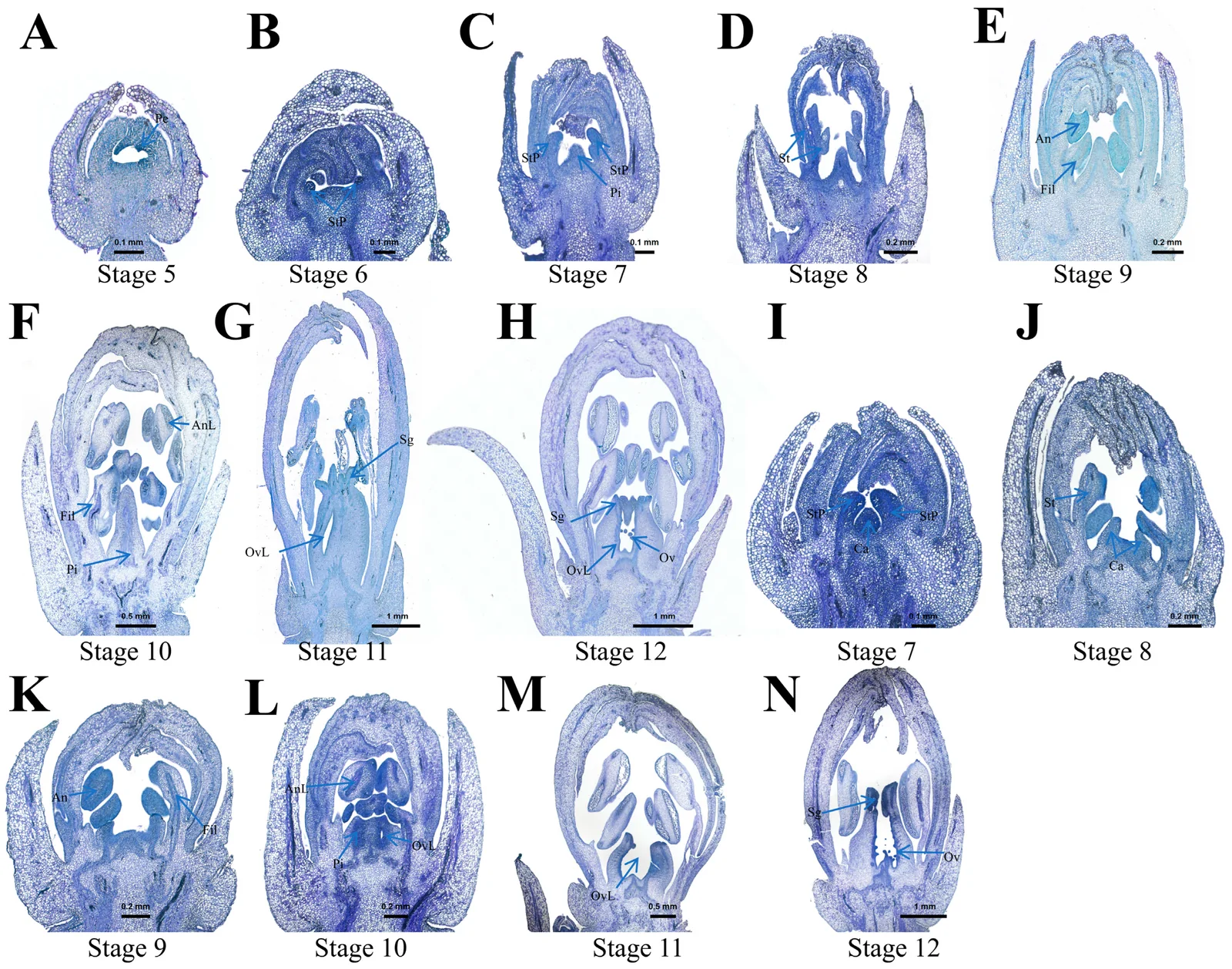
Histological characterization of Zhongbai hermaphroditic papaya flowers at different developmental stages. Abbreviations: Pe, petal; Ca, carpel; CP, carpel primordium; Pi, pistil; Sg, stigma; Ov, ovule; OvL, ovary locule; StP, stamen primordium; St, stamen; An, anther; AnL, anther locule; Fil, filament. (**A**) The petal structures began to form. (**B**) The sepal primordia emerged. (**C**) The ante-sepalous stamen primordia emerged, accompanied by the appearance of a rudimentary pistil. (**D**) The inner whorl of ante-petalous stamens emerged, resulting in the formation of two whorls of stamens. (**E**) The stamens developed complete anther and filament structures. (**F**) The anther locules gradually developed and matured, while the pollen mother cells progressively developed into pollen grains. (**G**) The five carpels gradually separated internally to form ovarian locules, while the apical region differentiated into the stigma. (**H**) The stigma matured, and ovules formed within the ovarian locules. (**I**) The carpel primordia emerged. (**J**) Two whorls of stamens emerged, while the carpels continued to develop. (**K**) The stamen structures began to differentiate. (**L**) The anther locules and ovary locules began to form. (**M**) The carpel apices developed further and differentiated into discoid structures. (**N**) The stigma developed further and matured, while ovules began to form within the ovary locules.

From Stage 5 onward, hermaphroditic flowers exhibited divergent pistil developmental fates and were classified into three major types based on the final outcome of pistil development. The first type developed both a functional pistil and fertile stamens. Early floral development (Stages 4–9) closely resembled that of male flowers, including corolla-tube formation, stamen initiation, anther differentiation, and the onset of pollen development (Figure 4A–E; Supplementary Figure S3F–H). However, unlike male flowers, the central carpel resumed female-like development from Stage 10 onward. At Stage 10, the pistil apex enlarged and differentiated into a stigma primordium, marking the transition from a rudimentary, nonfunctional pistil to a potentially fertile one. Meanwhile, the basal region of the pistil gradually expanded, initiating further pistil development (Figure 1T and Figure 4F). At Stage 11, the basal pistil continued to enlarge, and the internal tissues of the five fused carpels separated and differentiated to form five distinct ovary locules. During this process, the first divided into five lobes and subsequently followed a developmental trajectory similar to that of female flowers (Figure 4G; Supplementary Figure S3C,E). At Stage 12, numerous ovules developed within the ovary (Supplementary Figure S3I,J), and the characteristic antler-like stigma of papaya became fully developed, indicating that the hermaphroditic flower had reached full reproductive maturity before anthesis (Figure 4H; Supplementary Figure S3K).

A second developmental type also produced both a functional pistil and fertile stamens. However, unlike the first type, in which the central pistil initially exhibited a rudimentary developmental state and subsequently resumed female-like development, the pistil of the third type remained on a continuous fertile developmental trajectory from the early stages onward. The developmental patterns of the two types were similar at Stages 5 and 6, but became distinguishable with the initiation and subsequent development of the carpel primordia. At Stage 5, rapid petal elongation and outward expansion of the corolla base initiated corolla tube formation (Figure 4A). At Stage 6, the petals continued to develop, and five outer-whorl stamen primordia positioned opposite the sepals (antesepalous stamen primordia) emerged (Figure 4B). At Stage 7, five distinct carpel primordia became visible in the central region, indicating the early initiation of a potentially fertile pistil (Figure 4I). At Stage 8, five inner-whorl stamen primordia positioned opposite the petals (antepetalous stamen primordia) emerged, resulting in the formation of two whorls of stamens. At this stage, the developing stamens remained rod-like and showed no clear differentiation between anthers and filaments, while the five carpel primordia continued to enlarge and differentiate (Figure 4J). At Stage 9, the stamens underwent further differentiation, with the apical regions developing into anthers and the basal regions differentiating into filaments, resulting in clearly distinguishable anther and filament structures. Concurrently, the five carpel primordia continued to enlarge and progressively fused with one another (Figure 4K). At Stage 10, distinct anther locules began to form within the developing anthers, while the five carpels became closely fused and differentiated internally to form the ovary locules (Figure 4L). At Stage 11, the anther locules continued to develop and mature, accompanied by pollen development. Meanwhile, the apical region of the pistil further differentiated and expanded into discoid structures, whereas ovules were not yet detectable within the ovary locules (Figure 4M). At Stage 12, both the stamens and pistil reached maturity. The stigma became fully differentiated, ovules formed within the ovary locules, and the flower subsequently reached anthesis (Figure 4N).

Consequently, both floral types ultimately developed a functional pistil and fertile stamens. In contrast, the third type developed fertile stamens but an aborted pistil. Its development was largely similar to that of the first type through Stage 9. However, from Stage 10 forward, the central carpel structure failed to complete female differentiation. Incomplete carpel fusion or arrested internal tissue differentiation prevented the formation of normal ovary locules and ovules, whereas stamens developed normally and produced fertile pollen. As a result, the overall developmental trajectory of this floral type more closely resembled that of male flowers (Supplementary Figure S3J).

In addition to these two main types, two distinct developmental phenomena were observed in hermaphroditic flowers. The first was the recovery of an initially abortive pistil. In these flowers, a pistil that had reached an apparently mature but nonfunctional state re-entered the fertile pistil developmental program and resumed growth during Stages 8–10. Ultimately, the abortive pistil recovered and developed into a functional pistil. This transition was particularly evident shortly before anthesis.

The second phenomenon was stamen carpellody. In these flowers, abnormal carpel fusion occurred during early development, causing the five mature carpels to remain partially separated and form individual pistil-like structures. At the same time, the antesepalous stamens underwent varying degrees of degeneration and were progressively transformed into white, flattened carpellodic organs surrounding the central pistil. Based on the available developmental series, these abnormal organs arose through progressive stamen-to-carpel transformation (i.e., carpellody), in which normal stamens gradually acquired carpel identity and became incorporated into the developing pistil. Consequently, the typical hermaphroditic flower with two whorls of stamen was transformed into a hermaphroditic flower containing only a single functional stamen whorl (Supplementary Figure S4).

### 2.3. Global Transcriptomic Relationships Among Reproductive Organ States

To determine whether morphologically defined reproductive organ states were associated with distinct transcriptional programs, RNA-seq was performed on 33 samples representing 11 tissue groups, including normal pistils, aborted pistils, transitional pistils, carpellodic malformation complexes, and stamens. In the RNA-seq sample names, the prefixes E and M denote early and mature sampling classes, respectively, and are independent of the 12 morphological stages.

**Figure 5:**
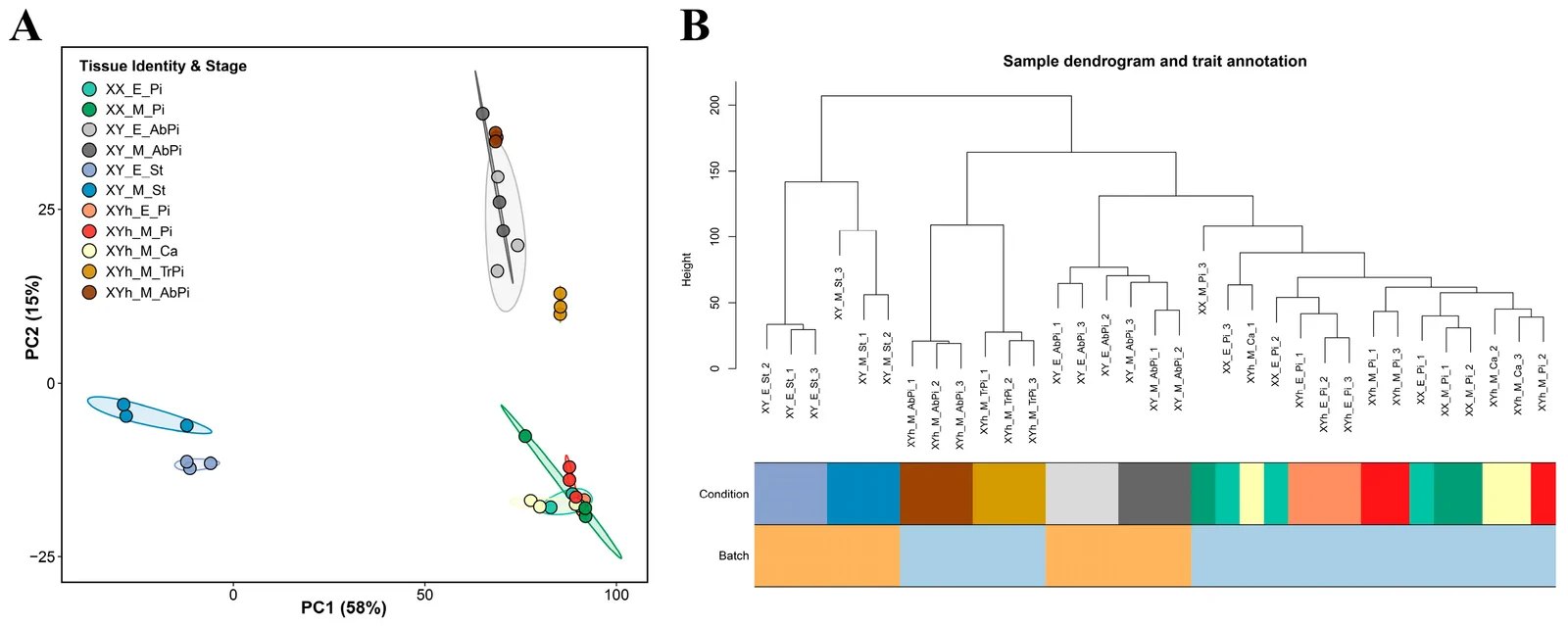
(**A**) Principal component analysis (PCA) plot of the 33 transcriptome samples. (**B**) Sample dendrogram and trait association heatmap.

Principal component analysis (PCA) based on the expression matrix of all genes showed that PC1 and PC2 explained 58% and 15% of the total variance, respectively (Figure 5A). Biological replicates clustered closely, indicating high reproducibility. Stamen samples were clearly separated from pistil and malformed-organ samples along PC1, whereas normal, transitional, and aborted pistils occupied distinct positions in the PCA space. These results indicate that morphologically distinct reproductive organ fates are associated with distinct gene expression profiles.

Hierarchical clustering produced a pattern broadly consistent with the PCA results (Figure 5B). The male stamen samples, XY_E_St and XY_M_St, formed a distinct branch, confirming their unique transcriptional identity. Within the pistil-related branch, aborted pistils from male and hermaphroditic flowers clustered adjacent to transitional pistils, suggesting shared transcriptional features associated with impaired pistil development. The carpellodic malformation complex, XYh_M_Ca, did not form a completely independent branch. Instead, one biological replicate clustered near normally developing female and hermaphroditic pistils, indicating that this tissue retained a substantial pistil-like transcriptional identity despite its abnormal morphology. Together these transcriptomic relationships among the different reproductive organ states suggested the existence of coordinated gene expression programs underlying their developmental divergence. We therefore performed WGCNA to identify co-expression gene modules associated with specific reproductive organ fates.

### 2.4. Identification of Key Modules Associated with Reproductive Organ Fate

**Figure 6:**
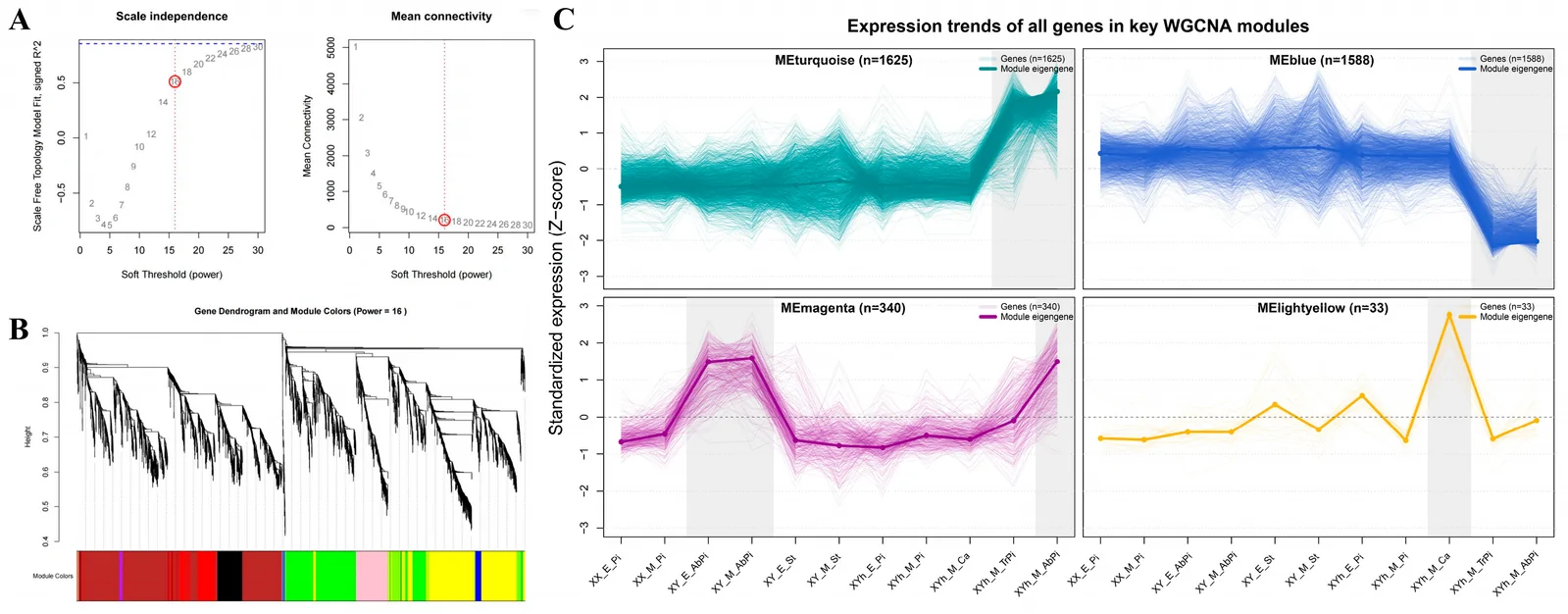
(**A**) Selection of the soft-thresholding power for WGCNA network construction. (**B**) Hierarchical clustering dendrogram for the identification of gene co-expression modules. (**C**) Expression trend analysis of all genes in the four core co-expression modules.

To identify gene networks associated with the developmental trajectories defined by morphological staging, WGCNA was performed using the filtered expression matrix. Based on module–trait correlations (Supplementary Figure S5) and expression patterns across samples (Supplementary Figure S6), four modules showing strong tissue or developmental specificity were selected for detailed analysis (Figure 6C).

The MElightyellow module contained 33 genes and was positively associated with the carpellodic malformation complex sample, XYh_M_Ca, exhibiting strong and localized upregulation in this tissue. The MEturquoise module comprised 1625 genes and was strongly associated with mature aborted pistils of hermaphroditic flowers (XYh_M_AbPi), with markedly elevated expression in this sample group. In contrast, the MEblue module, containing 1588 genes, was highly expressed in normally developing pistils but was downregulated in transitional and aborted pistils. The MEmagenta module contained 340 genes and was positively associated with aborted pistils of male flowers, particularly XY_E_AbPi and XY_M_AbPi, where its expression reached the highest levels.

Notably, the MEturquoise and MEmagenta modules were specifically associated with aborted pistils from hermaphroditic and male flowers, respectively, indicating that these two forms of pistil abortion are governed by distinct transcriptional programs.

To identify candidate regulatory genes, highly connected genes were extracted from each key module to construct module-specific co-expression networks (Supplementary Figure S7). Each network contained central hub genes with high connectivity and coordinated expression with neighboring genes, providing promising candidates for subsequent functional characterization.

### 2.5. Functional Characterization of Key Modules and Identification of Candidate Genes

**Figure 7:**
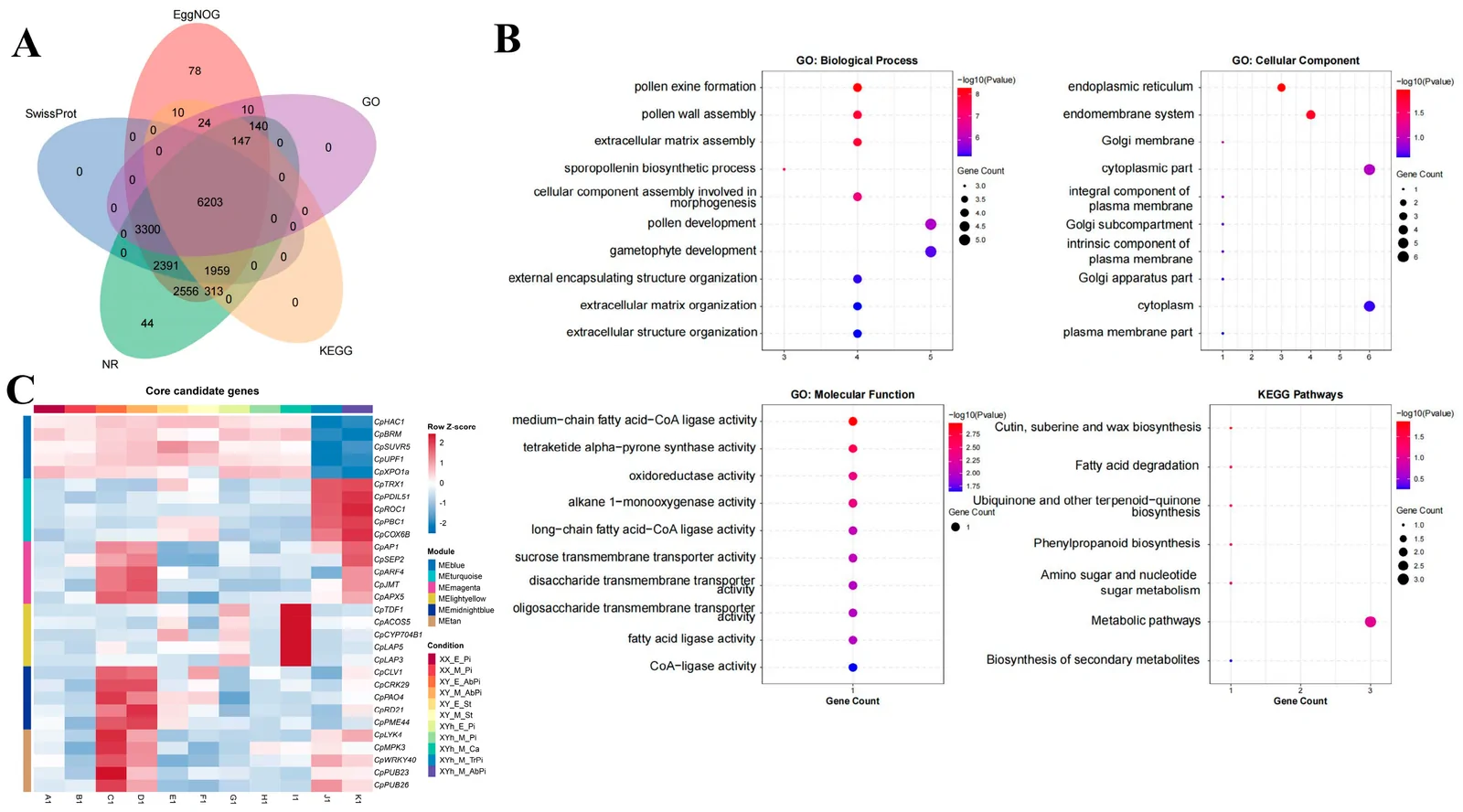
(**A**) Venn diagram of gene functional annotation across five databases; (**B**) KEGG and GO enrichment results of the core genes identified in the MElightyellow module; (**C**) heatmap of the 30 core candidate genes.

To characterize the biological functions associated with the key co-expression modules, GO and KEGG enrichment analyses were performed for six modules related to normal pistil development, pistil abortion, stamen carpellody, and signal-responsive processes. The modules showed distinct functional enrichment patterns (Figure 7; Supplementary Figures S8–S13).

The MEblue module, which was highly expressed in normally developing pistils, was enriched in chromatin organization, RNA processing, RNA degradation, mRNA surveillance, and nucleocytoplasmic transport. MEturquoise, associated with mature aborted pistils of hermaphroditic flowers, was mainly enriched in translation, ribosome biogenesis, oxidative phosphorylation, protein export, and protein processing in the endoplasmic reticulum. MEmagenta, associated with male pistillodes, was enriched in flower development, hormone response, signal transduction, transcriptional regulation, and vascular development.

The MElightyellow module was specifically enriched in pollen exine formation, pollen wall assembly, sporopollenin biosynthesis, gametophyte development, and lipid metabolism (Figure 7B). Although this module was associated with carpellodic floral complexes, its enrichment in male reproductive processes indicated that these tissues retained stamen-related molecular features. MEmidnightblue was enriched in developmental regulation, protein phosphorylation, plasma-membrane signaling, hormone signal transduction, and MAPK signaling, whereas MEtan was enriched in responses to chitin, biotic stimuli, stress, plant–pathogen interaction, MAPK signaling, and α-linolenic acid metabolism.

Based on module–trait associations, network connectivity, functional enrichment, and gene annotation, 30 representative candidate genes were selected from the six modules. Their expression profile showed clear module-specific patterns across normal pistils, transitional pistils, aborted pistils, stamens, and carpellodic tissues (Figure 7C). These candidates represent diverse biological processes, including chromatin and RNA regulation, protein homeostasis and metabolism, floral organ identity, hormone responses, pollen wall development, receptor-kinase signaling, MAPK signaling, stress responses, and ubiquitination-mediated regulation.

## 3. Discussion

### 3.1. Floral Staging Reveals the Early Morphological Node of Sex Differentiation in Papaya

This study established a 12-stage developmental system for male, female, and hermaphroditic papaya flowers from floral primordium initiation to anthesis. The three sex types were morphologically similar during Stages 1–4, when inflorescence meristem activation, floral primordium formation, sepal initiation, and petal initiation occurred. Visible divergence began at Stage 5, mainly through different developmental trajectories of the central reproductive region. Stage 5 can therefore be regarded as a key morphological starting point of sex differentiation in papaya.

This developmental framework also facilitates comparisons between papaya and established model systems. In Arabidopsis, AGAMOUS and other floral-organ identity regulators provide a molecular framework for stamen and carpel specification [20]. In cucumber, the arrest of male or female reproductive organs occurs within defined developmental windows [8]. Papaya differs from these systems because its carpel primordia may be initiated but subsequently maintain, lose, or recover their developmental competence. Therefore, the critical developmental event in papaya is not simply the presence or absence of carpel primordia, but whether these primordia can sustain growth, complete carpel fusion, and differentiate into functional ovary and stigma tissues. The early reduction in carpel growth in male flowers and some hermaphroditic flowers further suggests that pistil abortion is not exclusively a late degenerative event. Failure to complete carpel fusion, ovary-locule formation, placental differentiation, and stigma development is more consistent with an early failure to maintain gynoecial developmental competence. This interpretation predicts that the regulatory changes responsible for pistil suppression or abortion precede conspicuous tissue degeneration. Future spatial transcriptomic, hormone-profiling, and cell-type-specific analyses should therefore focus on the period immediately before and during Stages 5–7.

### 3.2. Pistil Developmental Competence Is a Core Morphological Indicator of Papaya Sex Differentiation

The most important morphological difference among papaya sex types was concentrated in the central pistil region. In female flowers and normal-pistil hermaphroditic flowers, five carpel primordia enlarged after Stage 5 and then underwent carpel fusion, ovary locule formation, placenta differentiation, ovule initiation, and stigma lobe formation. In contrast, male flowers and abortive-pistil hermaphroditic flowers formed only incomplete pistil-like structures and failed to establish a complete ovary and stigma. Therefore, pistil status should not be evaluated only by mature pistil size; instead, the critical criteria are whether carpel primordia enlarge synchronously, fuse properly, and continue to form ovary locules and stigma tissue after Stage 5.

This interpretation is consistent with previous transcriptomic studies suggesting that papaya pistil abortion is associated with altered expression of transcription factors and hormone-related genes, particularly auxin and brassinosteroid pathways [21]. Early sex differentiation in papaya has also been linked to transcriptional regulation, epigenetic regulation, plant hormones, and genes located in non-recombining regions of sex chromosomes [22]. The morphological divergence observed near Stage 5 may therefore represent the first visible output of earlier molecular differences.

Hermaphroditic flowers deserve special attention because they are not simply an intermediate or stable combination of male and female structures. Normal-pistil hermaphroditic flowers indicate that the female developmental program can be completed in the XYh background, whereas abortive-pistil hermaphroditic flowers retain stamen development but deviate from the normal pistil pathway early. The recovery of abortive pistils suggests that some tissues do not fully lose carpel developmental potential, and stamen carpellody indicates that organ identity boundaries may be unstable. These abnormalities should be interpreted cautiously as signs of altered coordination among pistil developmental competence, organ identity maintenance, and boundary control, rather than as evidence for a single causal gene or hormone. Environmental conditions may also influence this sensitivity. Low temperature can induce male-to-hermaphroditic floral conversion in papaya and is accompanied by changes in genes related to transcription factors, flower development, histone methylation, and hormone signaling [14]. Thus, the recovery of abortive pistils and stamen carpellody observed here may reflect the responsiveness of the hermaphroditic pistil developmental system to internal and external signals.

### 3.3. Morphologically Defined Reproductive Organ Fates Are Associated with Distinct Transcriptional Programs

The transcriptomic separation of normal pistils, transitional pistils, aborted pistils, male pistillodes, stamens, and carpellodic tissues supports the biological relevance of the morphology-based classification. More importantly, the module structure suggests that reproductive organs with superficially similar mature phenotypes do not necessarily follow the same molecular route. For example, the association of male pistillodes and aborted hermaphroditic pistils with different co-expression modules supports the developmental interpretation that persistent pistil suppression in XY flowers and pistil abortion in XYh flowers may represent partly distinct processes. These tissue types should therefore be analyzed separately in future mechanistic studies.

The association of MEblue with normally developing pistils suggests that sustained chromatin organization, RNA metabolism, and post-transcriptional regulation may contribute to the maintenance of gynoecial developmental competence. Its reduced expression in transitional and aborted pistils is consistent with a progressive loss of this competence. In contrast, the enrichment of translation, energy metabolism, redox regulation, protein folding, and protein turnover in MEturquoise suggests that pistil abortion in hermaphroditic flowers is accompanied by active metabolic and protein-homeostasis remodeling rather than by passive growth cessation alone. These interpretations remain hypotheses because WGCNA identifies correlations and cannot establish whether the modules are upstream regulators or downstream consequences of altered development.

The distinct association of MEmagenta with male pistillodes is consistent with the hypothesis that pistil suppression in male flowers differs from pistil abortion in hermaphroditic flowers. Although these tissues may appear morphologically similar at maturity, their different sex-chromosome backgrounds and module associations suggest that they reach an arrested state through partly different transcriptional routes. Future analyses should therefore compare these tissues at equivalent developmental stages and, where possible, within the same cultivar or closely related genetic backgrounds.

The pollen-wall formation, sporopollenin-biosynthesis, and lipid-metabolism signature associated with MElightyellow is compatible with the proposed stamen origin of the carpellodic structures. Retention of this male reproductive signature suggests that these organs do not undergo complete conversion into normal carpels but preserve aspects of their original stamen identity while acquiring carpel-like morphology. However, because the sampled carpellodic complexes may contain transformed stamens, normal stamens, and pistil tissues, spatial localization of candidate-gene expression will be required before these genes can be assigned specifically to the transformed cells.

The receptor-kinase, cell-wall, MAPK, WRKY, and ubiquitination-related functions represented by MEmidnightblue and MEtan provide possible links between intercellular signaling and reproductive-organ fate. Nevertheless, enrichment of stress- or immune-related pathways does not demonstrate that environmental stress directly determines floral-organ development. Many of these pathways also participate in general developmental signaling and cellular homeostasis. Controlled environmental experiments and tissue-specific functional analyses will therefore be required to distinguish developmental signaling from genuine stress responses.

Together, these findings support a working model in which sex-chromosome background constrains reproductive potential, whereas pistil developmental competence, organ-identity stability, and signal-responsive regulation influence the final reproductive-organ fate. This model generates three priorities for future research: resolving molecular changes immediately before and during Stages 5–7, localizing candidate-module expression to specific cell types, and functionally testing the representative candidate genes. An important limitation is that male and female flowers were sampled from cultivar AU9, whereas hermaphroditic flowers were sampled from cultivar Zhongbai. Consequently, cultivar effects cannot be fully separated from sex-type effects in comparisons involving hermaphroditic flowers. Validation using a common cultivar, additional genetic backgrounds, or near-isogenic materials will therefore be necessary before the identified transcriptional patterns can be considered broadly sex-specific or causal.

## 4. Materials and Methods

### 4.1. Plant Materials

Floral buds of *Carica papaya* L. were collected from hermaphroditic plants of the cultivar Zhongbai (ZB) and male and female plants of the cultivar AU9 during the peak flowering season. All plants were grown under greenhouse conditions at Fujian Agriculture and Forestry University, Fuzhou, China. Analyses focused on three flower types: AU9 male flowers, AU9 female flowers, and ZB hermaphroditic flowers.

For morphological and anatomical analyses, floral buds representing successive developmental stages were collected from floral meristem initiation to anthesis. Fresh buds were placed on ice immediately after collection and then fixed for microscopy and histological analysis. Buds were first grouped into ten size classes (0–1, 1–2, 2–3, 3–4, 4–5, 5–6, 6–7, 7–8, 8–9, and >10 mm), which served as preliminary references. Final staging was based on external morphology, internal anatomical features, and the chronological sequence of floral organ initiation and differentiation.

For transcriptome analysis, pistil-related tissues and stamens were micro-dissected under a stereomicroscope and immediately frozen in liquid nitrogen. The 11 RNA-seq sample groups are listed in Table 1. To avoid confusion with the 12 morphological stages defined in this study, RNA-seq samples were named using E and M to indicate early and mature sampling classes, respectively. The early class corresponded to 7–9 mm buds for female and hermaphroditic pistil-related tissues and 7–12 mm buds for male tissues, whereas the mature class corresponded to 15–20 mm buds. These RNA-seq sampling classes were based on bud size and dissected tissue status and should not be interpreted as equivalent to individual morphological stages.

### 4.2. Stereomicroscopic Observation and Morphological Staging

Fresh floral buds were examined with a Leica M205FA stereomicroscope (Leica Microsystems GmbH, Wetzlar, Germany). To reduce tissue desiccation during observation and dissection, samples were mounted on solid medium. Buds larger than 1 mm were held with fine forceps and dissected longitudinally to expose internal floral structures. For buds smaller than 1 mm, surrounding tissues were removed with a fine syringe needle to reveal the floral meristem and organ primordia.

Developmental stages were defined using bud size, overall morphology, floral organ initiation, and the relative developmental status of sepals, petals, stamens, and pistils, following the general principles of established floral staging systems [8,10]. Key landmarks included floral primordium formation, sepal initiation, petal initiation, stamen primordium emergence, carpel development, pistil differentiation, ovule formation, and anther maturation. Images were captured using the integrated imaging system of the stereomicroscope.

### 4.3. Histological Analysis and Paraffin Sectioning

Histological procedures followed standard plant microtechnique protocols with minor modifications. Floral buds were fixed in FAA solution containing absolute ethanol, formaldehyde, glacial acetic acid, and double-distilled water at a volume ratio of 10:2:7:1. Samples were vacuum-infiltrated for 1 h, fixed for an additional 24–48 h, and stored at 4 °C before processing.

Fixed buds were dehydrated through a graded ethanol series (50%, 60%, 70%, 80%, 85%, 95%, and 100%), cleared with xylene, and embedded in paraffin wax (56–58 °C) using a Leica EG1150H paraffin embedding station (Leica Biosystems Nussloch GmbH, Nussloch, Germany). Serial sections were cut at 7 μm using a Leica RM2255 rotary microtome (Leica Biosystems Nussloch GmbH, Nussloch, Germany), floated on a 42 °C water bath, mounted on poly-L-lysine-coated slides, and dried overnight at 42 °C. Sections were deparaffinized, rehydrated, stained with toluidine blue, rinsed, and observed under a light microscope.

Histological observations focused on floral meristem activity, floral organ primordium initiation, stamen and pistil differentiation, ovule development, pollen sac formation, and anatomical changes associated with floral organogenesis and sex differentiation.

### 4.4. Scanning Electron Microscopy

Samples for scanning electron microscopy (SEM) were prepared according to standard protocols for biological specimens with minor modifications [23]. Fixed and dehydrated floral buds were subjected to critical point drying, mounted on aluminum stubs, sputter-coated with gold, and observed using a Hitachi TM3030Plus scanning electron microscope (Hitachi High-Tech Corporation, Tokyo, Japan).

SEM was used to examine the three-dimensional morphology of floral primordia and developing organs, including sepal and petal primordia, stamen and pistil primordia, carpel fusion, stigma development, and anther surface morphology. SEM images were integrated with stereomicroscopic and histological observations to define the features of each developmental stage.

### 4.5. RNA Extraction and Transcriptome Sequencing

Total RNA was extracted from the collected floral tissues using the Magnetic Tissue/Cell/Blood Total RNA Kit (DP761; TIANGEN Biotech, Beijing, China), according to the manufacturer’s instructions. RNA concentration and purity were assessed using a NanoDrop One spectrophotometer (Thermo Fisher Scientific, Waltham, MA, USA), and RNA integrity was evaluated using an Agilent Fragment Analyzer 5400 system (Agilent Technologies Inc., Santa Clara, CA, USA). Only high-quality RNA samples were used for library construction.

Poly(A)-containing mRNA was enriched using the NEBNext^®^ Poly(A) mRNA Magnetic Isolation Module (New England Biolabs Inc., Ipswich, MA, USA). RNA-seq libraries were then constructed using the NEBNext Ultra II RNA Library Prep Kit for Illumina (New England Biolabs Inc., Ipswich, MA, USA). The libraries were sequenced on an Illumina NovaSeq X Plus platform in paired-end 150 bp mode. Each sample group contained three biological replicates, resulting in a total of 33 RNA-seq samples. Raw reads were processed with fastp (v0.23.4) to remove adaptors, low-quality reads, and reads with excessive unknown bases [24].

### 4.6. Quality Control, Read Alignment, and Expression Quantification

The RNA-seq experiment included 11 sample groups, including normal pistils, aborted pistils, transitional pistils, carpellodic floral complexes, male pistillodes, and stamens. In the sample names, XX, XY, and XYh indicate female, male, and hermaphroditic flowers, respectively; E and M indicate early and mature RNA-seq sampling classes; and Pi, AbPi, TrPi, Ca, Ps, and St indicate normal pistils, aborted pistils, transitional pistils, carpellodic complexes, male pistillodes, and stamens, respectively. These labels describe RNA-seq sampling classes and tissue types, not the 12 morphological developmental stages.

For each sample, a strand-specific paired-end library was constructed and sequenced by Personalbio, Nanjing, China, on an Illumina HiSeq 4000 Platform. Read filtering was performed with fastp using a base-quality threshold of Q20, a maximum low-quality base proportion of 30%, and no more than five ambiguous bases per read.

Clean reads were aligned to the papaya reference genome assembly GCA_021527605.1 (NCBI BioProject PRJNA727683) using HISAT2 (v2.2.1). The genome index was generated with hisat2-build, and —rna-strandness RF was specified for strand-specific libraries. SAM files were converted to sorted BAM files using SAMtools (v1.19.2). Gene-level read counts were quantified with featureCounts (v2.0.6; part of the Subread package) using the GWHBFSC00000000.gff annotation file from GWH project PRJCA006722. The merged count matrix was used for sample relationship analysis, differential expression analysis, and WGCNA.

Principal component analysis (PCA) was performed to assess sample relationships and biological replicate consistency. A DESeqDataSet object was generated in DESeq2 (v1.46.0; R/Bioconductor package) with design = ~1 for unsupervised analysis. Raw counts were transformed using variance stabilizing transformation (VST), and the plotPCA function was used to extract PC1 and PC2 coordinates and the variance explained by each component.

### 4.7. Construction of the WGCNA Co-Expression Network, Module Identification, and Gene Screening

Weighted gene co-expression network analysis (WGCNA) was performed using the WGCNA R package (v1.73) preprocessed expression matrix to identify modules associated with floral organ development, pistil abortion, transitional pistil formation, and carpellodic floral complexes. Sample groups were ordered as XX_E_Pi, XX_M_Pi, XYh_E_Pi, XYh_M_Pi, XYh_M_AbPi, XYh_M_TrPi, XYh_M_Ca, XY_E_Ps, XY_M_Ps, XY_E_St, and XY_M_St. Inter-sample distances were calculated from datExpr, and hierarchical clustering was performed with average linkage.

Candidate soft-thresholding powers included 1–10 and even numbers from 12 to 30. A soft-thresholding power of 16 was selected based on scale-free topology fit and mean connectivity. Networks were constructed with blockwiseModules using a signed network and signed topological overlap matrix (TOM). The minimum module size was 30 genes, modules with eigengene similarity greater than 0.75 were merged, and numeric labels were converted to WGCNA color labels.

A binary trait matrix was constructed for the 11 sample groups. Pearson correlations between module eigengenes and traits were calculated, and significance was assessed with Student’s *t*-test followed by Benjamini–Hochberg correction. Modules with |correlation| ≥ 0.5 and FDR < 0.05 were prioritized. For key module–trait pairs, module membership (MM) and gene significance (GS) were calculated, and candidate genes were selected using |MM| ≥ 0.8, |GS| ≥ 0.4, and consistent correlation direction between genes and module traits.

### 4.8. Screening of Core Differentially Expressed Genes and Functional Enrichment Annotation

Differential expression analysis was performed with DESeq2 using the raw gene-level count matrix. Genes with read counts ≥10 in at least three samples were retained. The 11 sample groups were treated as factor levels, and the design formula was set as ~ Condition. Differentially expressed genes (DEGs) were identified from 11 predefined pairwise comparisons. Genes with adjusted *p* value < 0.05 and |log2FoldChange| ≥ 1 were considered significant DEGs.

Hub genes from key WGCNA modules were intersected with relevant DEG lists to identify high-confidence candidates. Candidate genes were ranked using the following composite score: CandidateScore = |MM| × |GS| × |log2FoldChange| × [−log10(Padj)]. Genes with |MM| ≥ 0.8, |GS| ≥ 0.4, |log2FoldChange| ≥ 1, and Padj < 0.05 were retained for downstream annotation and visualization.

Protein sequences predicted from the reference genome were annotated using eggNOG-mapper (v2.1.12) in DIAMOND mode, DIAMOND (v2.1.9) blastp against Swiss-Prot, and NCBI BLAST+ (v2.16.0, blastp) blastp against the NCBI non-redundant plant protein database. Alignments used an E-value threshold of 1 × 10^−5^, and the best hit was retained for Swiss-Prot and NR searches. GO and KEGG enrichment analyses of WGCNA–DEG intersected gene sets were performed with clusterProfiler (v4.14.0; R/Bioconductor package), and enriched terms with *p* < 0.05 were considered significant.

Candidate genes were further mapped to Arabidopsis TAIR10 proteins using blastp (E-value < 1 × 10^−5^). Standardized papaya gene names were assigned using the prefix Cp. Genes with clear homologous annotations were named as Cp + gene symbol, whereas genes matching only a locus or predicted sequence were named conservatively. When multiple paralogs matched the same Arabidopsis target, numerical suffixes were added. A clustered heatmap of 30 representative core regulatory genes was generated from VST-transformed expression values using pheatmap (v1.0.12).

## 5. Conclusions

This study established a continuous 12-stage system for papaya flower development and identified Stage 5 as the earliest visible point of reproductive-organ divergence. Integration of morphological staging with RNA-seq and WGCNA distinguished transcriptional programs associated with normal pistils, male pistillodes, aborted and transitional hermaphroditic pistils, stamens, and carpellodic tissues, and identified 30 representative candidate genes involved in chromatin and RNA regulation, protein homeostasis, floral-organ identity, hormone and receptor-kinase signaling, pollen-wall formation, MAPK signaling, and ubiquitination. These findings provide a temporal framework and testable candidates for investigating papaya sex differentiation and reproductive developmental plasticity. Because male and female flowers were sampled from cultivar AU9 whereas hermaphroditic flowers were sampled from cultivar Zhongbai, sex-type and cultivar effects are partially confounded; validation across common or additional genetic backgrounds, together with spatial and functional analyses, will therefore be essential.

## Figures and Tables

**Table 1:** Sample information of floral organ tissues used in this study.

Group	Developmental Stage	Plant Sex Genotype	Sampled Tissue
XX_E_Pi	Early stage, 7–9 mm	XX	Pistil of female flower
XX_M_Pi	Mature stage, 15–20 mm	XX	Pistil of female flower
XYh_E_Pi	Early stage, 7–9 mm	XYh	Pistil of hermaphroditic flower
XYh_M_Pi	Mature stage, 15–20 mm	XYh	Pistil of hermaphroditic flower
XYh_M_AbPi	Aborted stage, 15–20 mm	XYh	Aborted pistil of hermaphroditic flower
XYh_M_TrPi	Transitional stage, 15–20 mm	XYh	Transitional pistil of hermaphroditic flower
XYh_M_Ca	Abnormal stage, 15–20 mm	XYh	Carpelized abnormal complex
XY_E_St	Early stage, 7–12 mm	XY	Stamen of male flower
XY_M_St	Mature stage, 15–20 mm	XY	Stamen of male flower
XY_E_AbPi	Early stage, 7–12 mm	XY	Aborted pistil of male flower
XY_M_AbPi	Mature stage, 15–20 mm	XY	Aborted pistil of male flower

## Data Availability

The raw RNA-sequencing data generated in this study have been deposited in the Genome Sequence Archive (GSA) at the National Genomics Data Center (NGDC), China National Center for Bioinformation, under accession number PRJCA069298. The processed gene expression matrix and other data supporting the findings of this study are available in the Supplementary Materials or from the corresponding author upon reasonable request.

## Supplementary Materials

The following supporting information can be downloaded at https://www.mdpi.com/article/10.3390/plants15162530/s1: Figure S1: Paraffin sections of AU9 male papaya flowers at different developmental stages; Figure S2: Histological characterization of AU9 female papaya flowers at different developmental stages; Figure S3: Histological characterization of Zhongbai hermaphroditic papaya flowers at different developmental stages; Figure S4: Stereomicroscopic images of pistil carpellody during the development of hermaphroditic papaya flowers; Figure S5: Module–trait relationship heatmap of WGCNA; Figure S6: Module eigengene expression patterns across reproductive organ developmental states; Figure S7: Co-expression networks of the four key WGCNA modules; Figure S8: Completeness of functional annotation across five public databases; Figure S9: GO and KEGG enrichment analyses of hub genes in the MEblue module; Figure S10: GO and KEGG enrichment analyses of hub genes in the MEturquoise module; Figure S11: GO and KEGG enrichment analyses of hub genes in the MEmagenta module; Figure S12: GO and KEGG enrichment analyses of hub genes in the MEmidnightblue module; Figure S13: GO and KEGG enrichment analyses of hub genes in the MEtan module.

## Abbreviations

The following abbreviations are used in this manuscript:

AbPi	Aborted pistil
An	Anther
AnL	Anther locule
BAM	Binary Alignment Map
Ca	Carpellodic complex
CP	Carpel primordium
DEG	Differentially expressed gene
FDR	False discovery rate
Fil	Filament
GO	Gene Ontology
GS	Gene significance
KEGG	Kyoto Encyclopedia of Genes and Genomes
MAPK	Mitogen-activated protein kinase
MM	Module membership
NCBI	National Center for Biotechnology Information
NR	NCBI non-redundant protein database
Ov	Ovule
OvL	Ovary locule
PCA	Principal component analysis
Pe	Petal
PeP	Petal primordium
Pi	Pistil
Ps	Pistillode
Rec	Receptacle
RNA-seq	RNA sequencing
SAM	Sequence Alignment/Map
Se	Sepal
SEM	Scanning electron microscopy
SeP	Sepal primordium
Sg	Stigma
St	Stamen
StP	Stamen primordium
TAIR	The Arabidopsis Information Resource
TOM	Topological overlap matrix
TrPi	Transitional pistil
VST	Variance stabilizing transformation
WGCNA	Weighted gene co-expression network analysis
XX	Female sex genotype
XY	Male sex genotype
XYh	Hermaphroditic sex genotype
