# Supplementary material for: Morphological Staging and Transcriptomic Analyses Reveal Divergent Reproductive Organ Fates in Papaya Flowers
Source: Plants (Basel). 2026 Aug 21;15(16):2530. doi: 10.3390/plants15162530 (PMC13516351; doi:10.3390/plants15162530)
Supplement: Supplementary file 1 [file plants-15-02530-s001.zip › plants-4473188-supplementary.pdf]

Supplementary Materials

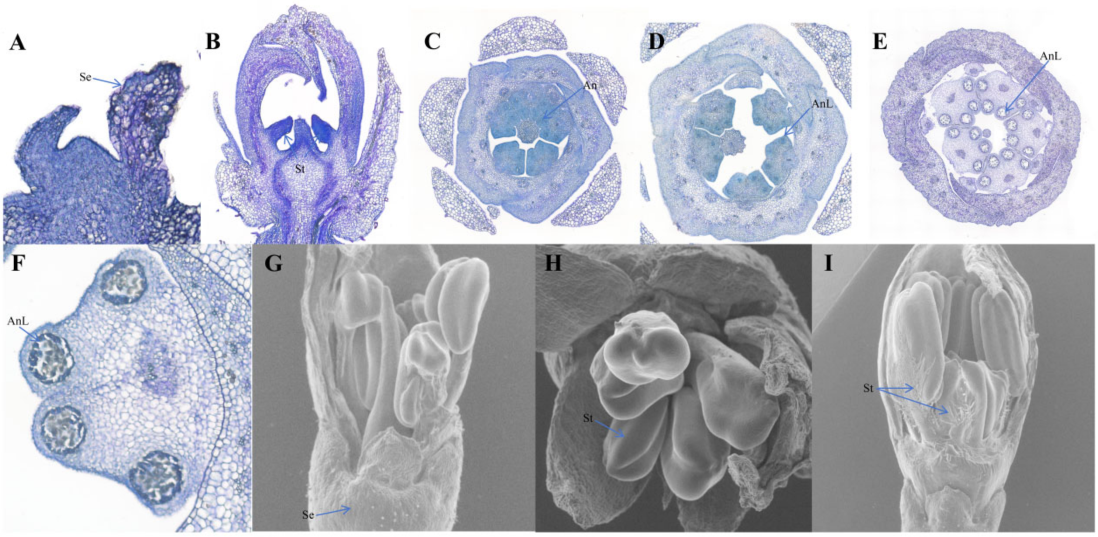

Supplementary Figure S1. Paraffin sections of AU9 male papaya flowers at different developmental stages. Abbreviations: Se, sepal; Pe, petal; St, stamen; An, anther; AnL, anther locule.(A) Further development of the sepal primordia. (B) Sepal primordia further developed and gradually enclosed the floral primordium, while petal primordia were initiated.(C) The ante-sepalous stamen primordia elongated and formed stalk-like structures.(D) Transverse section of a male flower showing the initial formation of anther locules. (E) Transverse section of a male flower showing the appearance of pollen mother cells within the anther locules. (F) Transverse section of a male flower showing mature anther locules. (G) Transverse section of a mature anther locule. (H) SEM showing the overall structure of a male flower.(I) SEM of the outer whorl of stamens.(J) SEM showing the two whorls of stamens.

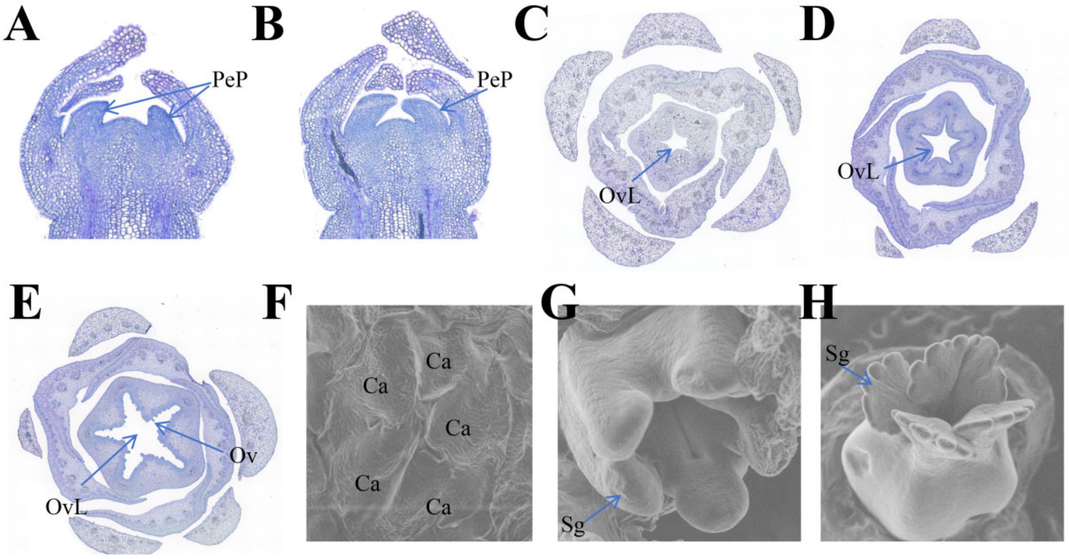

Supplementary Figure S2. Histological characterization of AU9 female papaya flowers at different developmental stages.Abbreviations: PeP, petal primordium; Pe, petal; Ca, carpel; Sg, stigma; Ov, ovule; OvL, ovary locule. (A) Initiation of petal primordia. (B) Further elongation of petal primordia into

stalk-like structures. (C) Transverse section showing newly formed ovary locules without ovules. (D) Transverse section showing the initiation of ovules within the ovary locules. (E) Transverse section showing mature ovary locules containing numerous ovules.(F) Complete fusion of the five carpels.(G) Differentiation of the carpel apices into disc-shaped structures.(H) Formation of a fully developed antler-like stigma

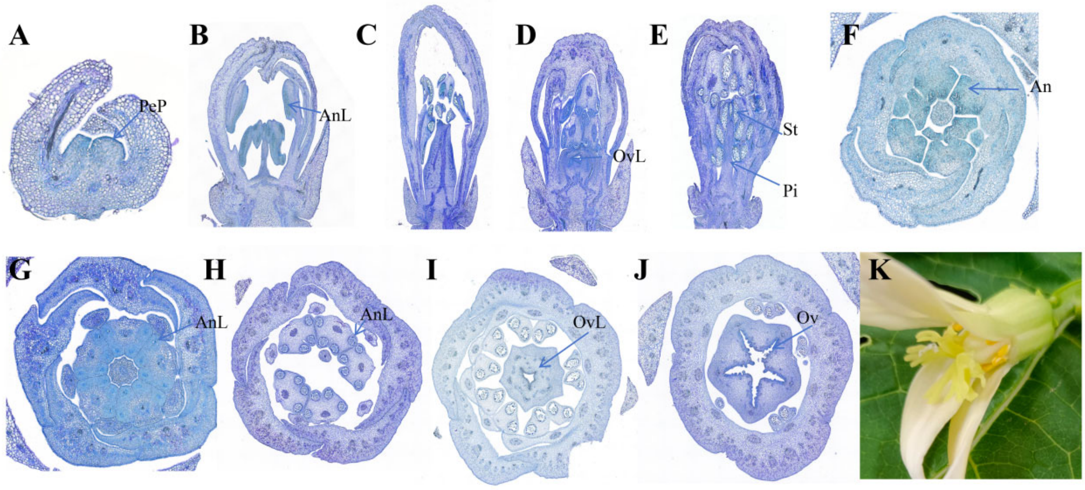

Supplementary Figure S3. Histological characterization of Zhongbai hermaphroditic papaya flowers at different developmental stages.Abbreviations: Pe, petal; CP, carpel primordium; Ca, carpel; Pi, pistil; Sg, stigma; Ov, ovule; OvL, ovary locule; StP, stamen primordium; St, stamen; An, anther; AnL, anther locule; Fil, filament; Rec, receptacle.(A) The petal precursor structures were visible.(B) Anther locules began to form.(C) The pistil, composed of five carpels, rapidly enlarged.(D) Differentiation of the ovarian locules was completed.(E) A bisexual flower containing a rudimentary pistil.(F) Transverse section of a bisexual flower showing anthers in which anther locules had not yet formed.(G) Transverse section of a bisexual flower showing the initial formation of anther locules.(H) Transverse section of a bisexual flower showing mature anther locules.(I) Transverse section of a bisexual flower showing ovarian locules before ovule formation.(J) Transverse section of a bisexual flower showing ovules within the ovarian locules.(K) A blooming bisexual flower.

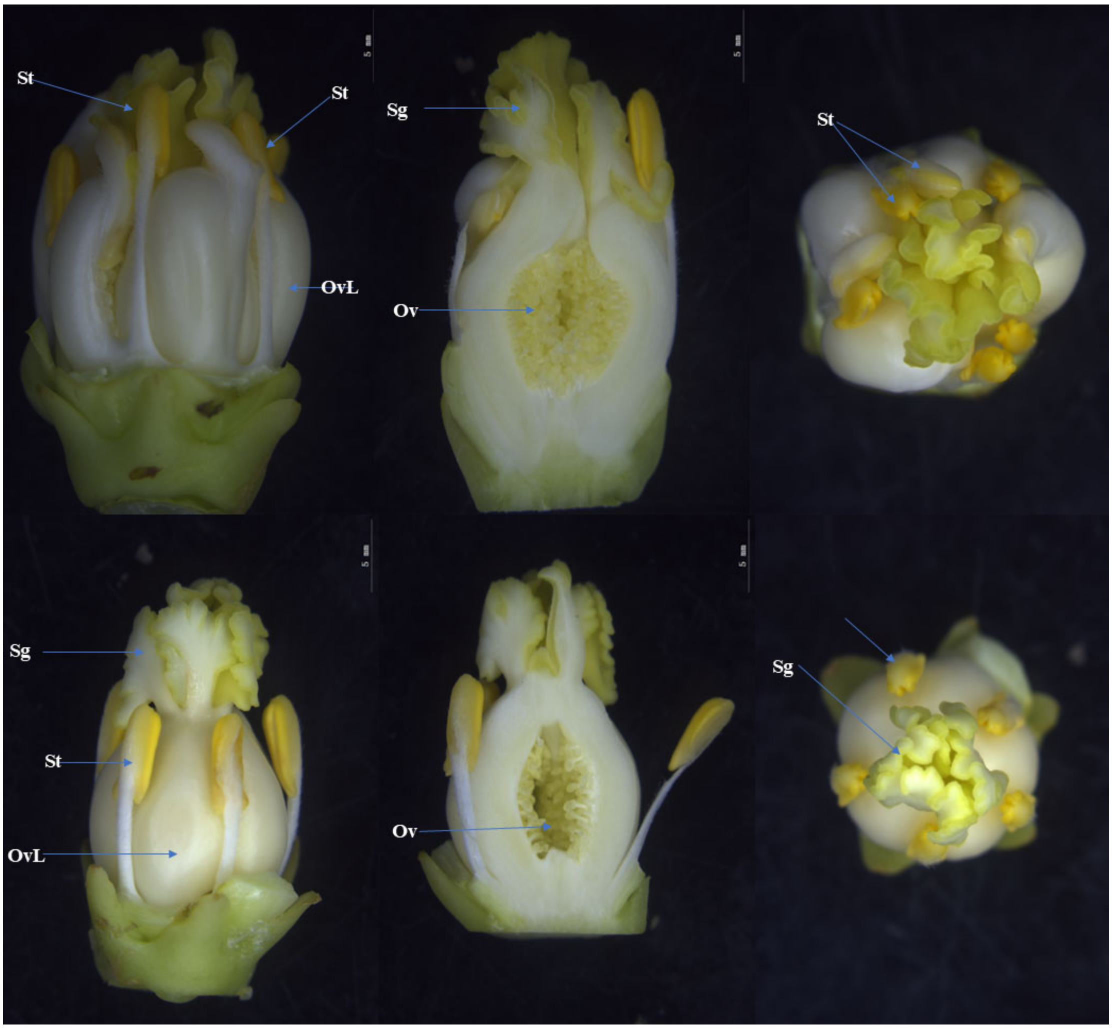

Supplementary Figure S4. Stereomicroscopic images of pistil carpellody during the development of hermaphroditic papaya flowers.

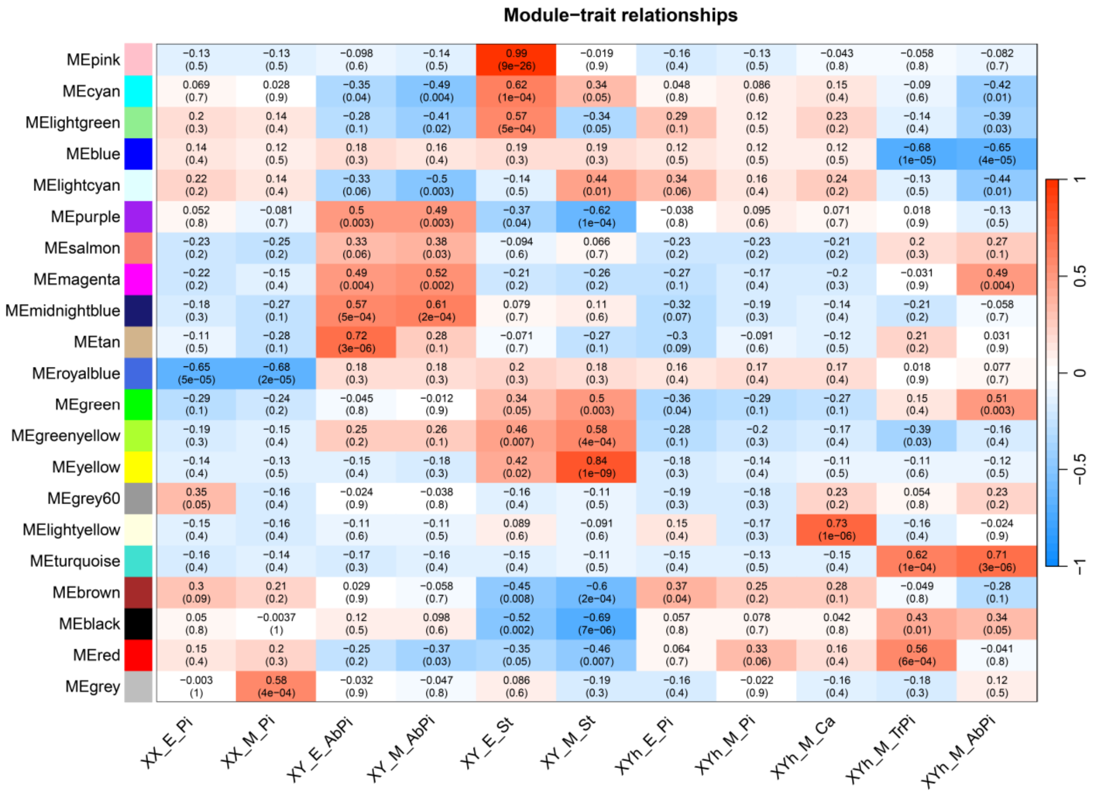

Supplementary Figure S5. Module–trait relationship heatmap of WGCNA.

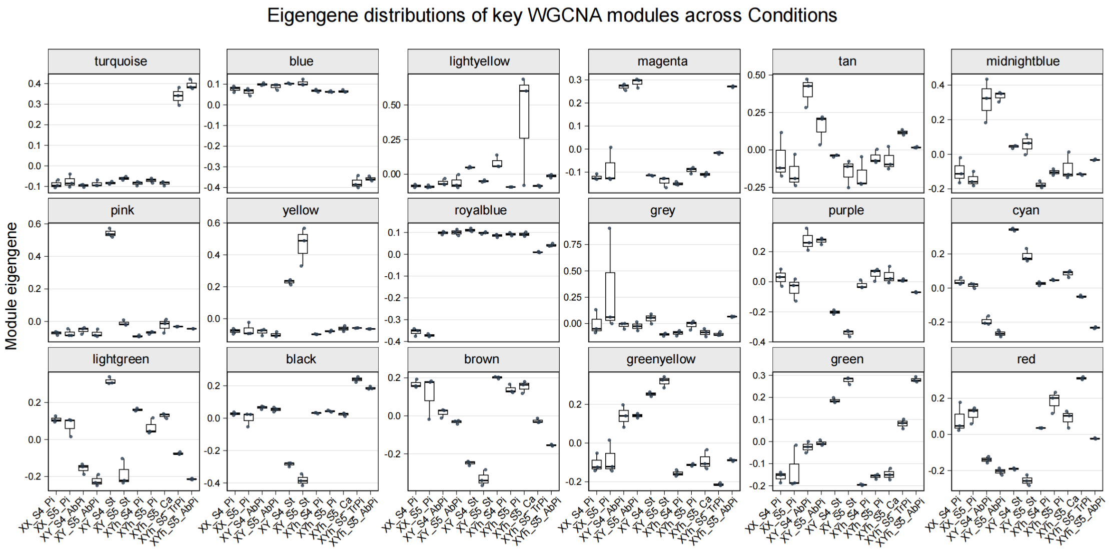

Supplementary Figure S6. Module eigengene expression patterns across reproductive organ developmental states.

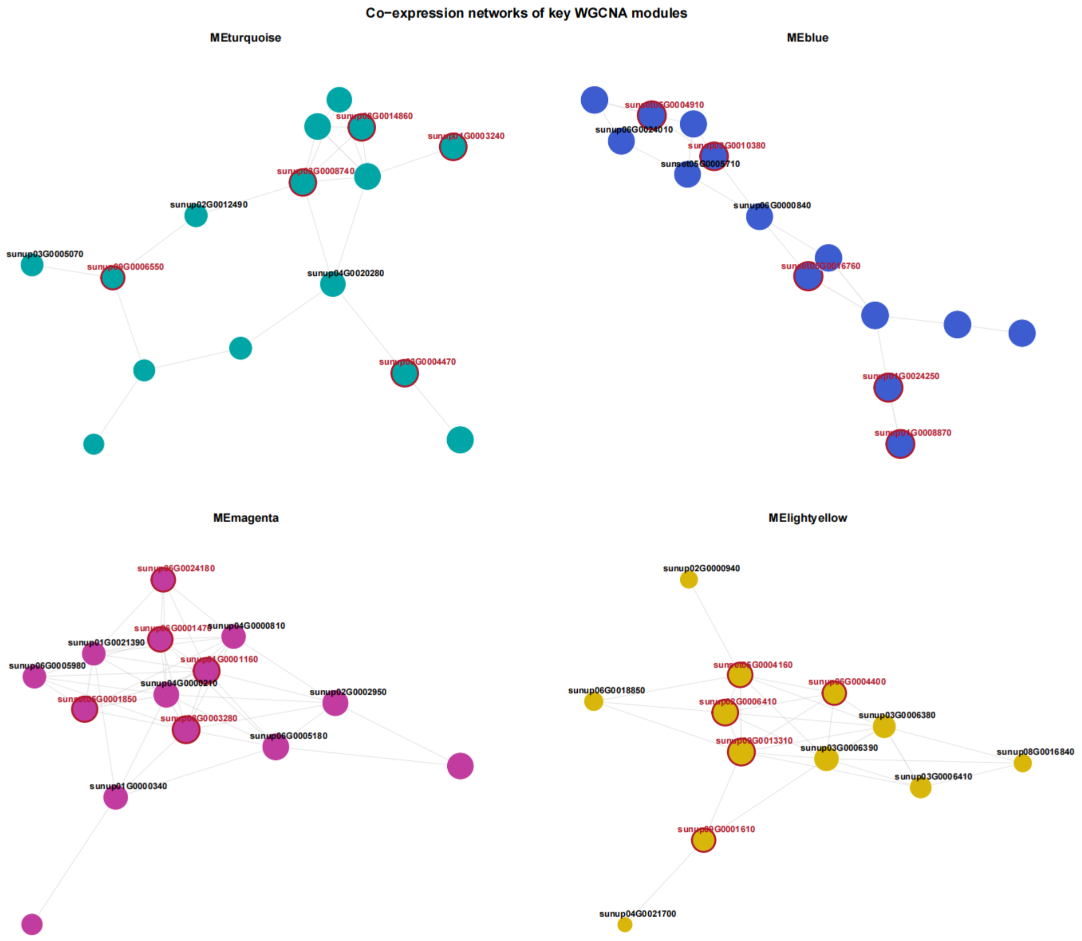

Supplementary Figure S7. Co-expression networks of the four key WGCNA modules.

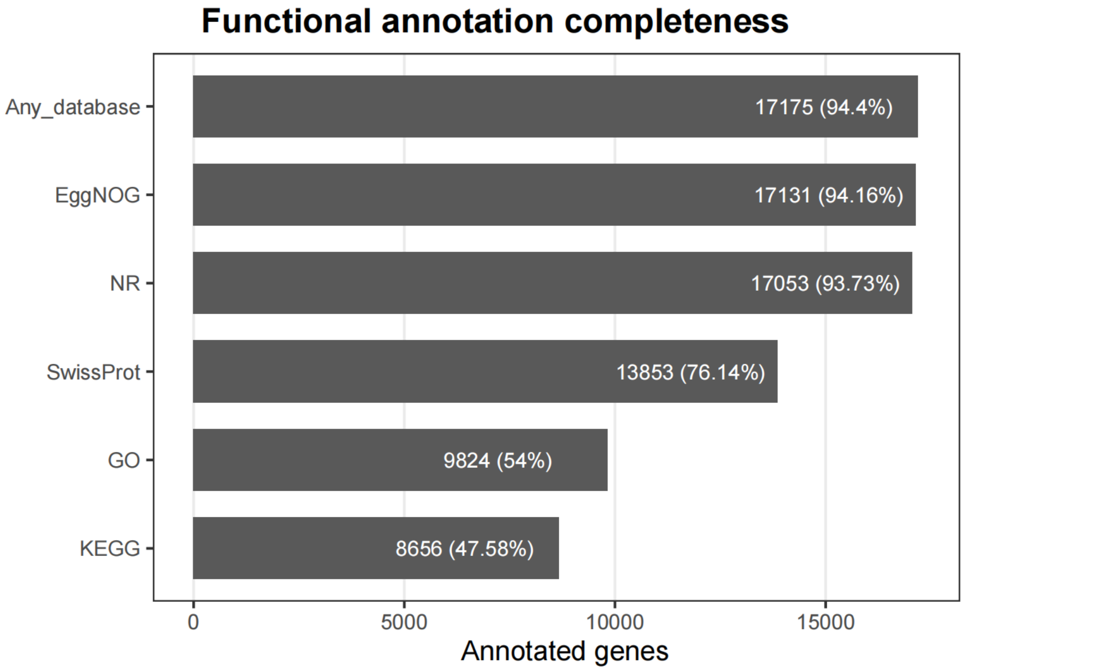

Supplementary Figure S8. Completeness of functional annotation across five public databases.

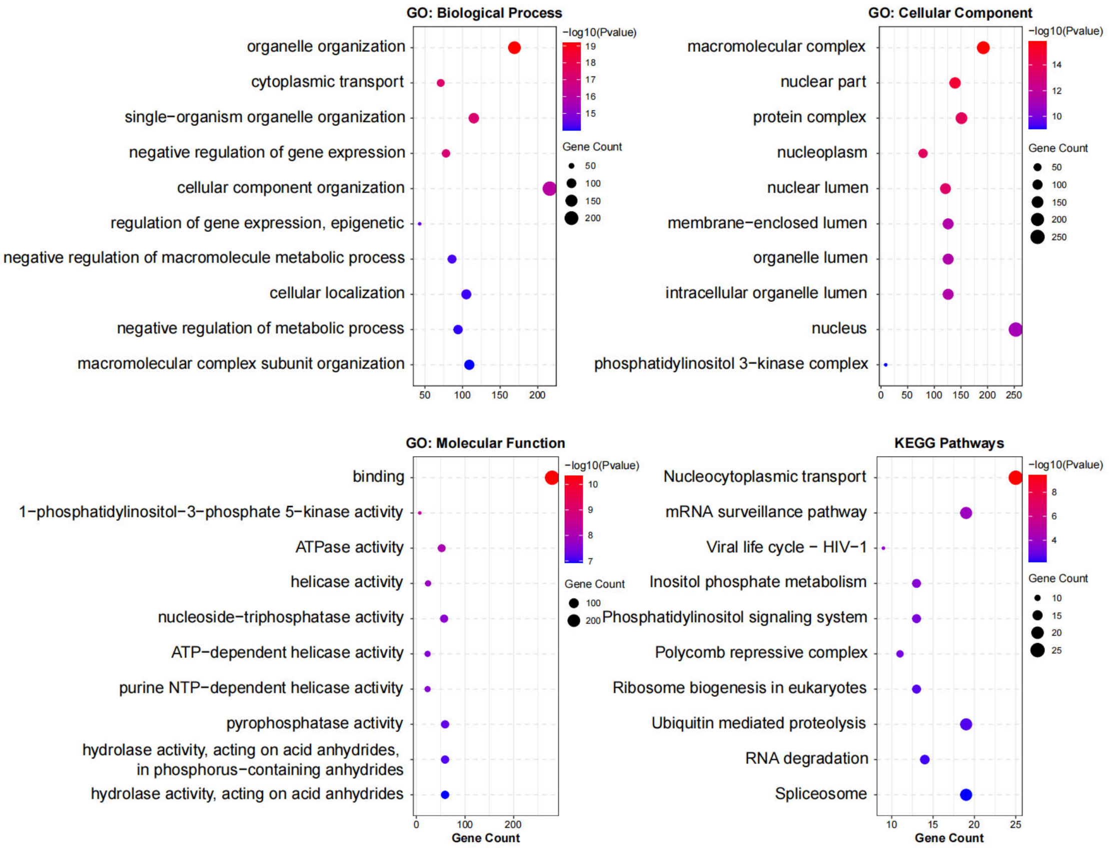

Supplementary Figure S9. GO and KEGG enrichment analyses of hub genes in the MEblue module.

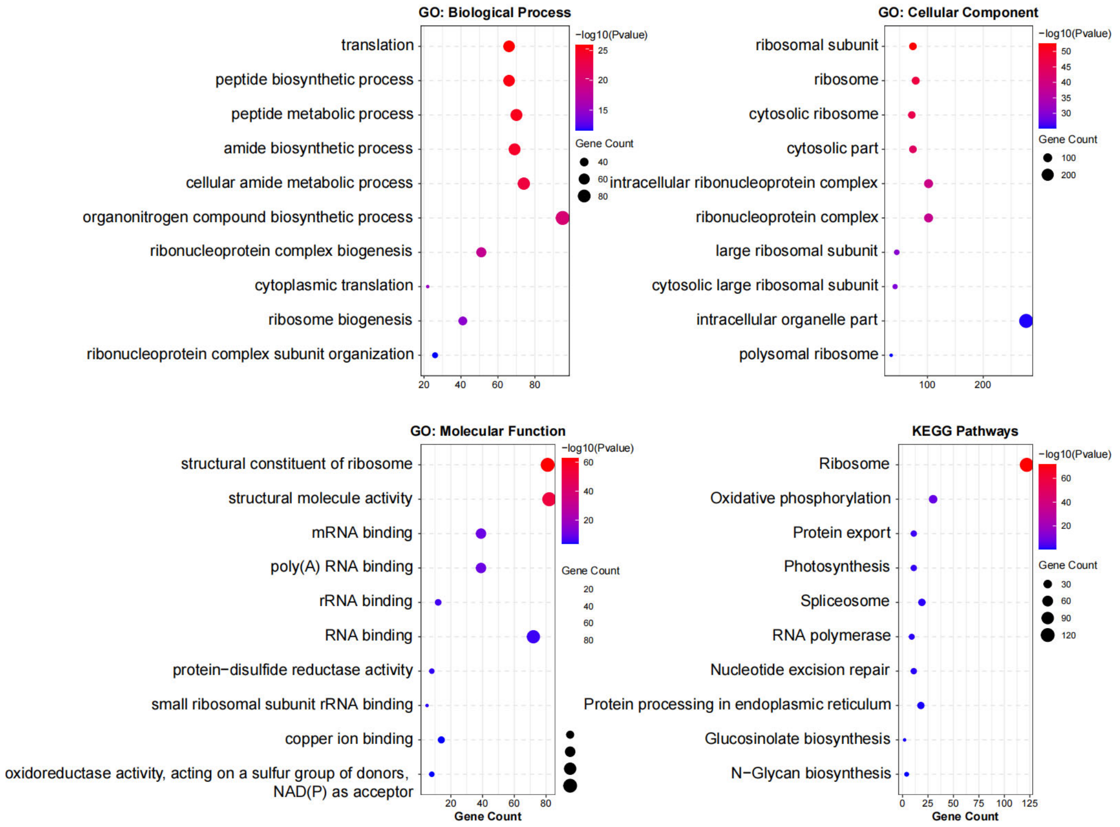

Supplementary Figure S10. GO and KEGG enrichment analyses of hub genes in the MEturquoise

module.

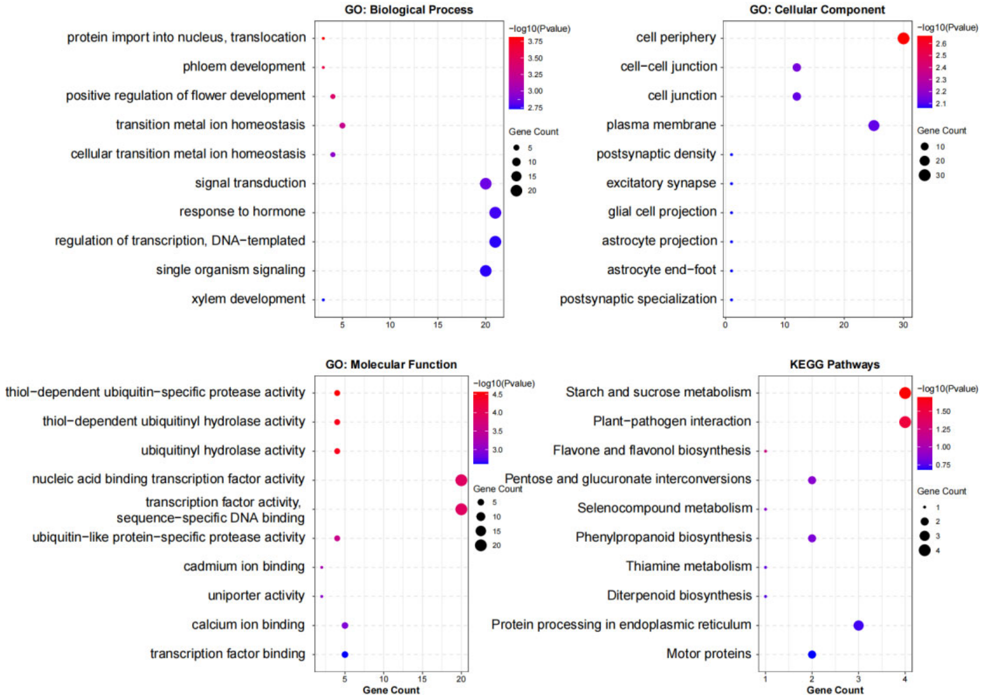

Supplementary Figure S11. GO and KEGG enrichment analyses of hub genes in the MEmagenta module.

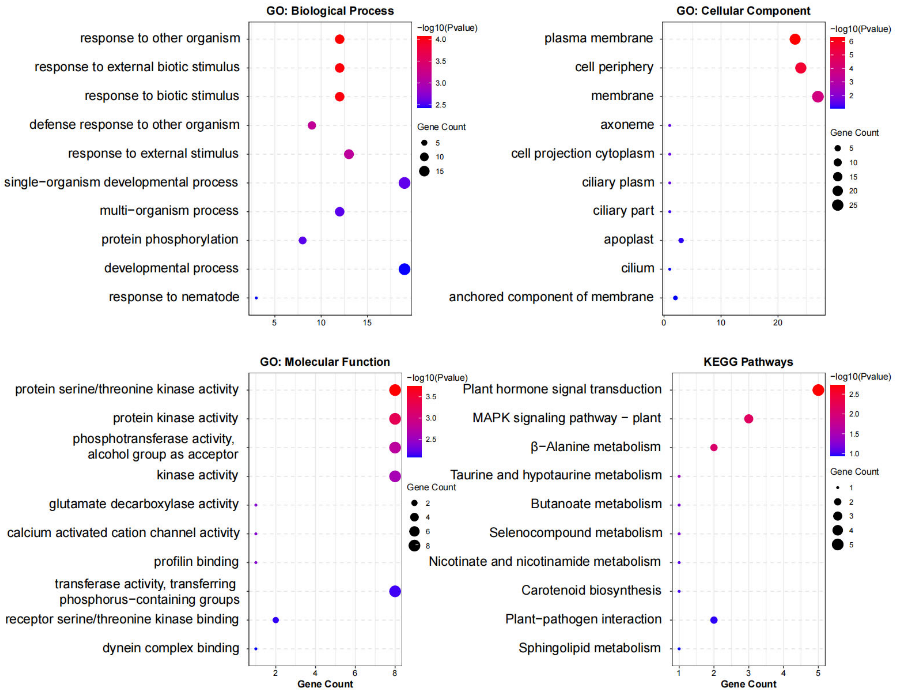

Supplementary Figure S12. GO and KEGG enrichment analyses of hub genes in the MEmidnightblue module.

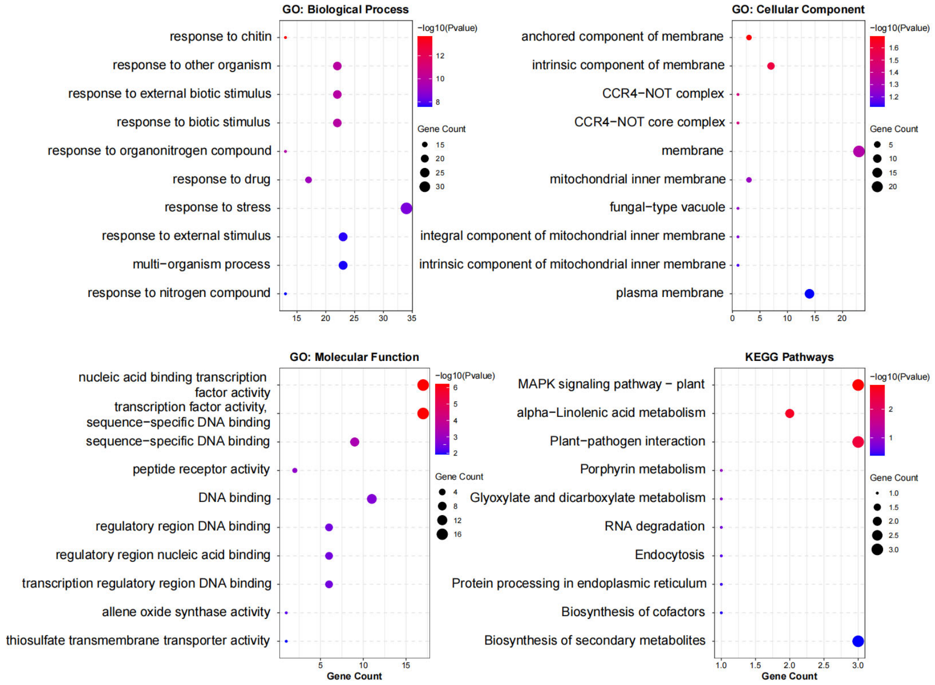

Supplementary Figure S13. GO and KEGG enrichment analyses of hub genes in the MEtan module.
